# Development of a humanized mouse model with functional human materno-fetal interface immunity

**DOI:** 10.1172/jci.insight.176527

**Published:** 2024-10-22

**Authors:** Shuai Dong, Cong Fu, Chang Shu, Min Xie, Yan Li, Jun Zou, Yi-Zi Meng, Peng Xu, Yan-Hong Shan, Hui-Min Tian, Jin He, Yong-Guang Yang, Zheng Hu

**Affiliations:** 1 Key Laboratory of Organ Regeneration & Transplantation of Ministry of Education, Department of Obstetrics, Obstetrics and Gynaecology Center, The First Hospital of Jilin University, Changchun, China.; 2 National-Local Joint Engineering Laboratory of Animal Models for Human Diseases, Changchun, China.; 3 International Center of Future Science, Jilin University, Changchun, China.

**Keywords:** Immunology, Reproductive biology, Adaptive immunity, Mouse models, Obstetrics/gynecology

## Abstract

Materno-fetal immunity possesses specialized characteristics to ensure pathogen clearance while maintaining tolerance to the semiallogeneic fetus. Most of our understanding on human materno-fetal immunity is based on conventional rodent models that may not precisely represent human immunological processes owing to the huge evolutionary divergence. Herein, we developed a pregnant human immune system (HIS) mouse model through busulfan preconditioning, which hosts multilineage human immune subset reconstitution at the materno-fetal interface. Human materno-fetal immunity exhibits a tolerogenic feature at the midgestation stage (embryonic day [E] 14.5), and human immune regulatory subsets were detected in the decidua. However, the immune system switches to an inflammatory profile at the late gestation stage (E19). A cell–cell interaction network contributing to the alternations in the human materno-fetal immune atmosphere was revealed based on single-cell RNA-Seq analysis, wherein human macrophages played crucial roles by secreting several immune regulatory mediators. Furthermore, depletion of Treg cells at E2.5 and E5.5 resulted in severe inflammation and fetus rejection. Collectively, these results demonstrate that the pregnant HIS mouse model permits the development of functional human materno-fetal immunity and offers a tool for human materno-fetal immunity investigation to facilitate drug discovery for reproductive disorders.

## Abstract 2

Pregnant humanized mouse is a unique model to facilitate the investigation of human reproductive immunology and relevant reproductive diseases.

## Introduction

Fetuses express major histocompatibility complex (MHC) antigens from both parents. Thus, the exposure of paternally derived semiallogeneic antigens on fetal tissues to the maternal immune system may evoke antifetus immune responses and result in reproductive disorders or even fetal loss under certain conditions (1). Specialized anatomical structures and the immune atmosphere are developed at the materno-fetal interface to control potential immune responses and ensure the exchange of nutrients or metabolic elements (2). Increasing evidence illustrates that the immune system is not under a static immune inhibition or antiinflammatory status at the materno-fetal interface (3); instead, it is delicately regulated and experiences dynamic alternation from embryo implantation to fetal delivery (4, 5). Errors occurring in this process may lead to reproductive complications such as recurrent spontaneous abortion, preeclampsia, and intrauterine growth restriction (6–8).

The human materno-fetal immune system exhibits a distinct immune profile in the immune subset composition/phenotype or cellular function compared with its counterpart in blood and lymphoid organs (9, 10). One prominent difference is a large enrichment of natural killer (NK) cells that occupy approximately 70% of all immune subsets during the first trimester in decidual tissues (11). In addition, these decidual NK (dNK) cells have specific phenotypes (such as upregulated expression of CD49a and CD103) and functions (such as immune modulation and facilitating fetal development) (8, 12, 13). The proportion of decidual macrophages that exhibit the immune-inhibitory phenotype (upregulated CD206 expression) also increases. These cells specialize in secreting several cytokines (such as TGF-β) and chemokines (such as CCL2 and CXCL9) that characterize the immune atmosphere (14). Adaptive immune subsets occupy a relatively smaller proportion at the materno-fetal interface, whereas the counts of regulatory T (Treg) cells (a subset playing key roles in immune tolerance induction) are markedly elevated (15). An impairment in human Treg cell number or function is closely corelated to pregnancy disorders (16).

Currently, a major part of our knowledge regarding the development and function of the materno-fetal interface immune system is based on conventional rodent models. However, many differences exist between the rodent and human immune systems (17). For instance, mouse and human T cells host diverse expression patterns for some key costimulatory molecules (such as CD28), and some cytokines (such as IFN-γ) may play an opposite role in the pathological process of rodents and humans (18). Therefore, it is uncertain whether the outcome and principle built on mouse experiments can be used to explain human reproductive immunological processes during pregnancy and if the drugs or therapies that are effective in conventional rodent tests for treating reproductive complications can be clinically translated. Recently, deep phenotyping has provided a high-resolution view of human pregnancy biology from early to late gestation and placenta or basal plate research (19–22). However, these results primarily offer static descriptions. The dynamic developmental processes underlying maternal and fetal tolerance remain unclear. Thus, there is an urgent need to develop an in vivo model capable of directly recapitulating human materno-fetal immunity formation and operation.

Humanized mouse models are rodents incorporated with human genes, cells, or tissues that represent human physiological and pathological characteristics and connect bench discovery with clinical application (23). Our previous studies and those of others demonstrate that the Thy/HSC human immune system (HIS) humanized mouse (also called the BLT model; ref. 24), generated by transplanting human fetal thymic tissue (beneath the renal capsule) and human CD34^+^ fetal liver cells (intravenous injection) after sublethal total body irradiation (TBI), hosts high levels of multilineage human lymphoid and hematopoietic cell reconstitution in immunodeficient mice (25, 26). Potent antigen-specific T cell responses and human IgM and IgG production were examined in Thy/HSC mice after immunization, allogeneic/xenogeneic transplantation, or pathogen challenge. Humanized mice have been applied in several biomedical fields (27–31). However, whether they can be utilized to study human materno-fetal immunity remains unknown.

Here, we revealed that a conventional humanized mouse model generated using the TBI protocol cannot give birth after mating and developed a humanized pregnant mouse model based on busulfan preconditioning, wherein multilineage, specialized human immune subsets reconstitute at the mouse materno-fetal interface. Moreover, the reconstructed human materno-fetal immunity is functional. In sum, we explored the application of a humanized mouse model in human reproductive immunology and offer a tool for the evaluation of preclinical therapeutic intervention.

## Results

### Establishment of a pregnant HIS mouse model.

**Figure 1:**
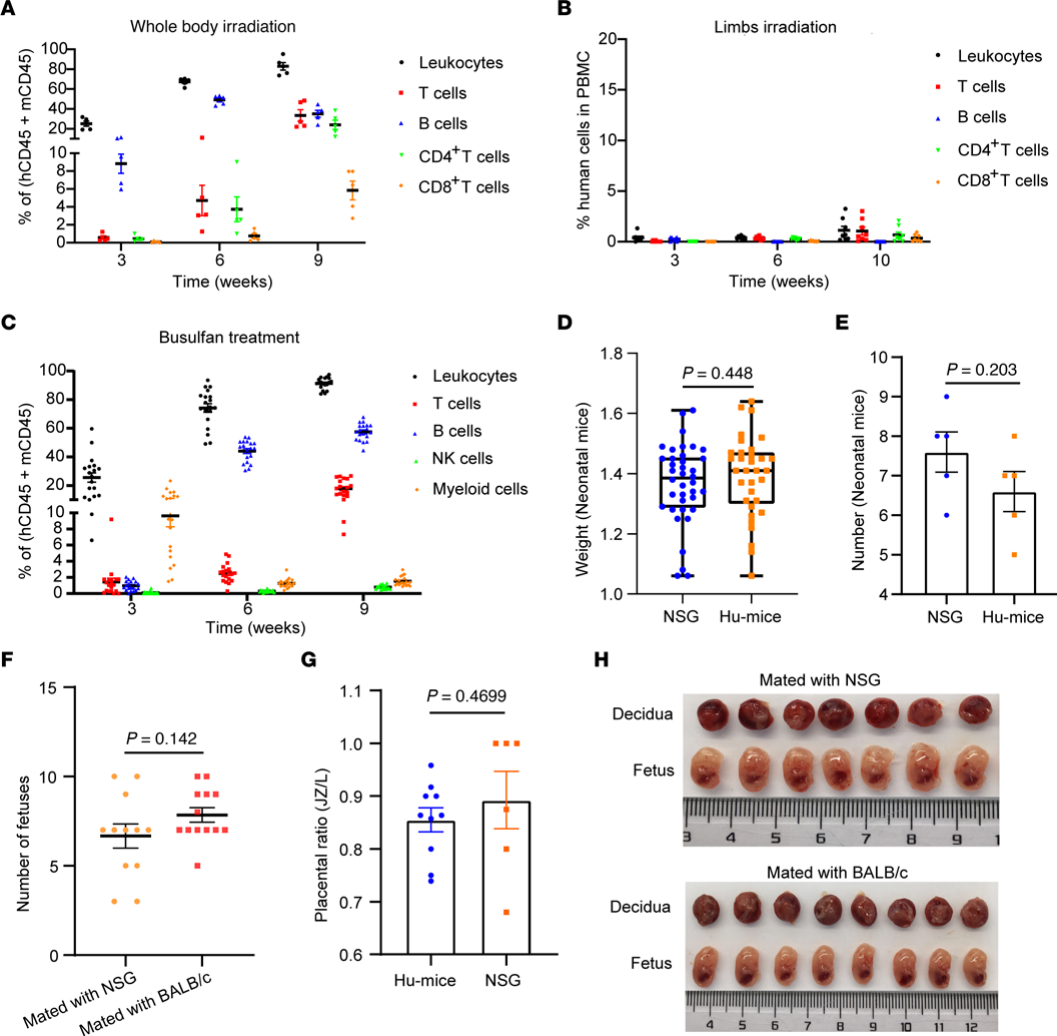
Construction of a pregnant HIS mouse model with high levels of human lymphohematopoietic cell reconstitution. (**A**–**C**) Ratios (mean ± SEM) of multilineage human lymphohematopoietic cell reconstitution within human CD45^+^ plus mouse CD45^+^ cell population of HIS mice made by intravenous injection of human CD34^+^ fetal liver cells and transplantation of human fetal thymic tissue under the renal capsule after preconditioning by TBI (**A**; *n* = 5), limb local irradiation (**B**; *n* = 8), or busulfan treatment (**C**; *n* = 19). Percentages of human CD45^+^ leukocytes, CD3^+^ T cells, CD19^+^/CD20^+^ B cells, NK cells, and CD33^+^ myeloid cells in PBMCs at indicated weeks were shown. An unpaired *t* test was used to analyze the differences in the ratios of human CD45^+^ cells, T cells, and B cells in the PBMCs at week 9 between the TBI and busulfan groups: CD45^+^ cells: *P* < 0.01; T cells: *P* < 0.001; B cells: *P* < 0.0001. (**D**) The average weight of neonatal mice born to NSG or HIS mice that were mated with BALB/c male mice (busulfan treatment, *n* = 5). Box plots show the interquartile range, median (line), and minimum and maximum (whiskers). (**E**) The number of neonatal mice born by NSG or humanized mice that were mated with BALB/c male mice (busulfan treatment, *n* = 5). (**F**) The average fetus number (mean ± SEM) of pregnant HIS mice made by mating with NSG males (*n* = 12) or BALB/c males (*n* = 13) summarized (busulfan treatment) at E14.5. (**G**) Ratio of placental junctional to labyrinth zone (JZ/L ratio) in humanized mice (hu-mice) (*n* = 10) and NSG mice (*n* = 6) on E14.5. (**H**) Pictures of placentas and fetuses of a representative HIS mouse euthanized at E14.5 were shown. Statistical analyses were performed using unpaired *t* test (**D**–**G**).

We first examined whether Thy/HSC HIS mice generated using the conventional protocol can be used for the human materno-fetal immunity study. Six- to 8-week-old female NSG mice were preconditioned with sublethal TBI, followed by transplantation of human fetal thymic tissue (under the renal capsule) and intravenous injection of human CD34^+^ fetal liver cells (24, 32, 33). High levels of multilineage human lymphohematopoietic cells were detected in peripheral blood mononuclear cells (PBMCs) of the mice from week 6 after humanization (Figure 1A); this was consistent with our previous reports (32). However, these HIS mice failed to get pregnant after mating with NSG males for 2 months. This was presumably due to the TBI treatment, which causes severe damage to mouse reproductive capabilities (34, 35). Therefore, we conditioned the mice by limb local irradiation to construct the HIS mouse model to avoid irradiating the reproductive organs. All 5 HIS mice got pregnant and delivered healthy pups after mating with NSG males; however, all mice had very low levels (<1.5%) of human lymphohematopoietic cells (Figure 1B).

After several tests, we found that HIS mice generated using the busulfan-based preconditioning protocol, a bifunctional alkylating agent that kills proliferating hematopoietic stem/progenitor cells in recipients by crosslinking guanine bases in DNA double-helix strands when used as a myeloablative regimen before hematopoietic stem cell transplantation (36), with less toxicity against reproductive organs (37), have high levels of human lymphohematopoietic cells, composed of reconstituted human CD3^+^ T cells, CD20^+^ B cells, CD56^+^ NK cells, and CD33^+^ myeloid cells (Figure 1C). The HIS female mice efficiently got pregnant and delivered healthy pups. A comparable number and weight of well-developed neonatal mice were observed between the HIS mice and NSG mice after mating with BALB/c males (Figure 1, D and E). In addition, the fetus number of HIS female mice after mating with NSG or BALB/c males was comparable at E14.5 (Figure 1, F and H). There was no significant difference in the ratio of placental junctional to labyrinth zone between HIS mice and NSG mice (Figure 1G). No fetal demise or resorption events were observed in busulfan-treated pregnant mice. These data demonstrate that pregnant HIS mice can be constructed by busulfan preconditioning.

### Composition of multilineage human immune subsets at mouse materno-fetal interface.

**Figure 2:**
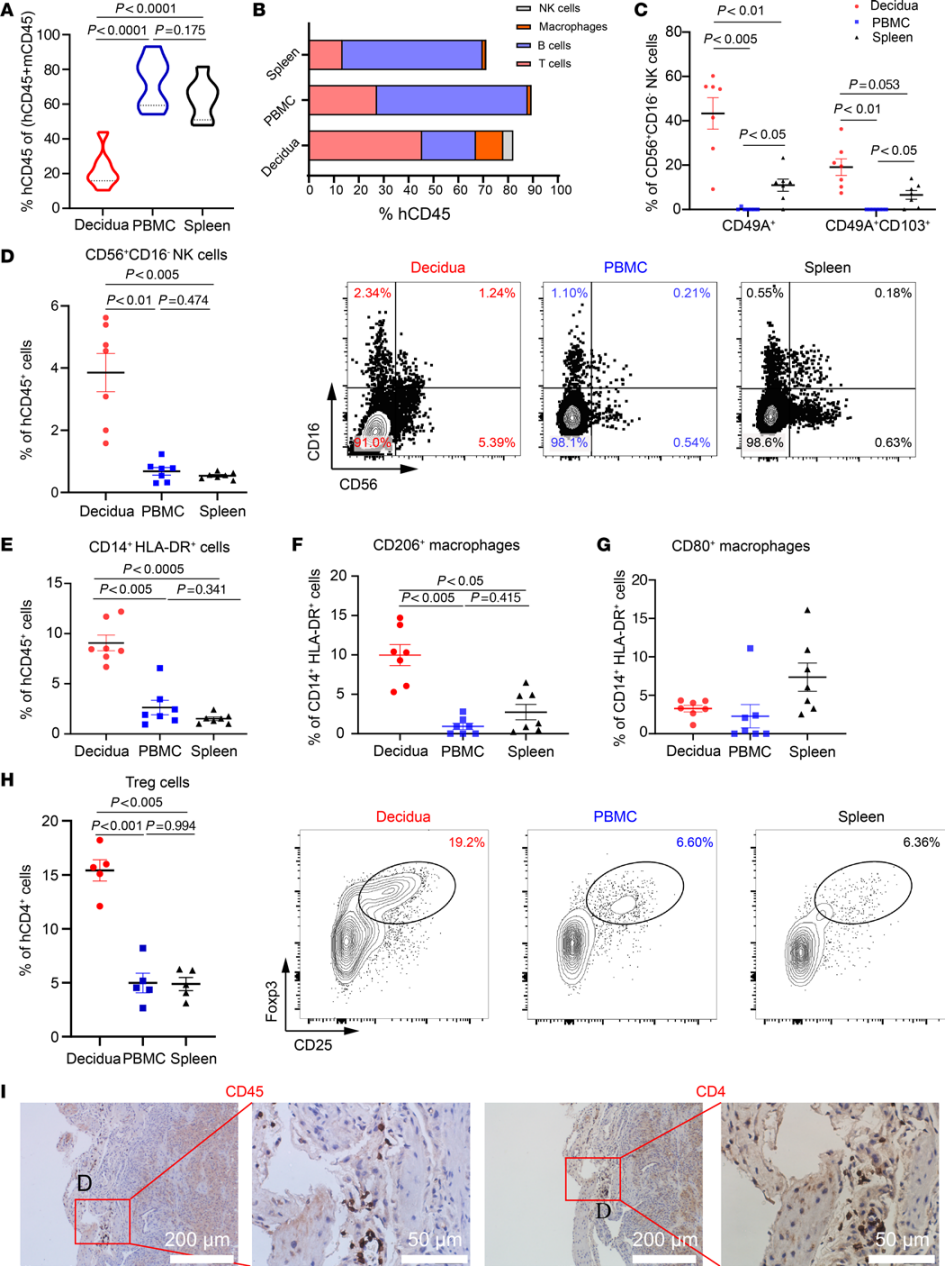
Human immune subset composition in pregnant HIS mice at E14.5. HIS mice (*n* = 10) made by the busulfan protocol were mated with BALB/c males and euthanized at E14.5 for analysis. Summarized data about the chimerism (mean ± SEM) of human lymphohematopoietic cells (**A**) (%huCD45^+^/[%hCD45^+^ + %mCD45^+^]) and ratios (**B**) of CD3^+^ T cells, CD20^+^ B cells, CD14^+^ macrophages, and CD56^+^ NK cells within human CD45^+^ cells in PBMCs, spleen, and decidua were shown. (**C**) Summarized results (mean ± SEM; *n* = 7) of the percentages of CD49a^+^ and CD49a^+^CD103^+^ tissue-resident NK cells in human CD56^+^CD16^–^ NK cells. (**D**) Summarized results (mean ± SEM; *n* = 7) of the percentages of CD56^+^CD16^–^ NK cells and representative flow cytometric profiles were shown. (**E**–**G**) Summarized results (mean ± SEM; *n* = 7) of the percentages of CD14^+^HLA-DR^+^ human macrophages (**E**), CD206^+^ M2 macrophages (**F**), and CD80^+^ M1 macrophages (**G**) were shown. (**H**) Summarized results (mean ± SEM; left; *n* = 5) and representative flow cytometry (FCM) profiles (right) of the ratios of Treg cells. (**I**) Immunohistochemical (IHC) examination of human CD45^+^ lymphohematopoietic cells (left) and CD4^+^ T cells (right) in decidua. D, decidua. Statistical differences were determined with 1-way ANOVA for multiple-variable comparisons (**A** and **C**–**H**).

Pregnant HIS mice were euthanized at E14.5 after mating with syngeneic NSG or allogeneic BALB/c males to examine the human immune cell composition and phenotype at decidua. Multilineage human immune cells were detected in the mouse decidua, PBMCs, and spleen under either mating condition (Figure 2, A and B, and Supplemental Figure 1, A and B; supplemental material available online with this article; https://doi.org/10.1172/jci.insight.176527DS1). The vast majority were T and B cells, and only small populations were macrophages and especially NK cells. While human CD14^+^HLA-DR^+^ macrophages and CD56^+^ NK cells were markedly enriched in the decidua compared with that in the PBMCs and spleen (Figure 2, D and E, and Supplemental Figure 1, D and E), similar to what is observed in humans. Human maternal immune cells at the decidua are naturally exposed to semiallogeneic paternally derived antigens (38). Therefore, our study focused on pregnant HIS mice made by mating with BALB/c males in the following experiments.

Immune subsets at the materno-fetal interface have specialized phenotypes and functions (39). We found an approximately 7-fold higher number of CD56^+^CD16^–^ NK cells (a specific NK subset that secretes cytokines) in the decidua compared with that in the PBMCs and spleen (Figure 2D). Moreover, markedly higher levels of CD49a and CD103 (2 molecules that play crucial roles in retaining tissue characteristics and can be used to identify human dNK cells) were detected in human NK cells in the decidua than in the PBMCs and spleen (Figure 2C). Additionally, human immune subsets with potent immune-modulatory function were significantly enriched at the materno-fetal interface. Higher ratios of immunosuppressive M2 macrophages (CD206^+^), but not inflammatory M1 macrophages (CD80^+^), were observed in human CD14^+^HLA-DR^+^ macrophages in the decidua than those in the PBMCs or spleen (Figure 2, F and G, and Supplemental Figure 1, E–G). Furthermore, significantly higher ratios of CD25^+^Foxp3^+^CD4^+^ Treg cells (the immune subset playing key roles in immune tolerance induction and maintenance) were found in the decidua than in the PBMCs and the spleen (Figure 2H). Marked reduction of CD45RA^+^CCR7^+^ naive T cell and elevation of CD45RA^–^CCR7^+^ effector memory T cell (CD4^+^ and CD8^+^ T cells) counts were observed in the decidua (Supplemental Figure 2, B and C, and Supplemental Figure 3, A and B), which appeared to acquire an experienced and differentiated phenotype. Interestingly, the proportions of decidua human CD49a^+^ dNK, CD49a^+^CD103^+^ dNK, and CD25^+^Foxp3^+^CD4^+^ Treg cells were markedly higher under allogeneic mating conditions than under syngeneic mating conditions (Figure 2, C and H; Supplemental Figure 1C; and Supplemental Figure 2A). IHC examination of HIS mouse decidua samples further substantiated the distribution of human CD45^+^ lymphohematopoietic cells and human CD4^+^ T cells at the decidua (Figure 2I). These data demonstrate that pregnant HIS mice host a specialized HIS at the materno-fetal interface.

### Tolerogenic to inflammatory conversion of the materno-fetal immune profile across the mid to late gestation stages.

**Figure 3:**
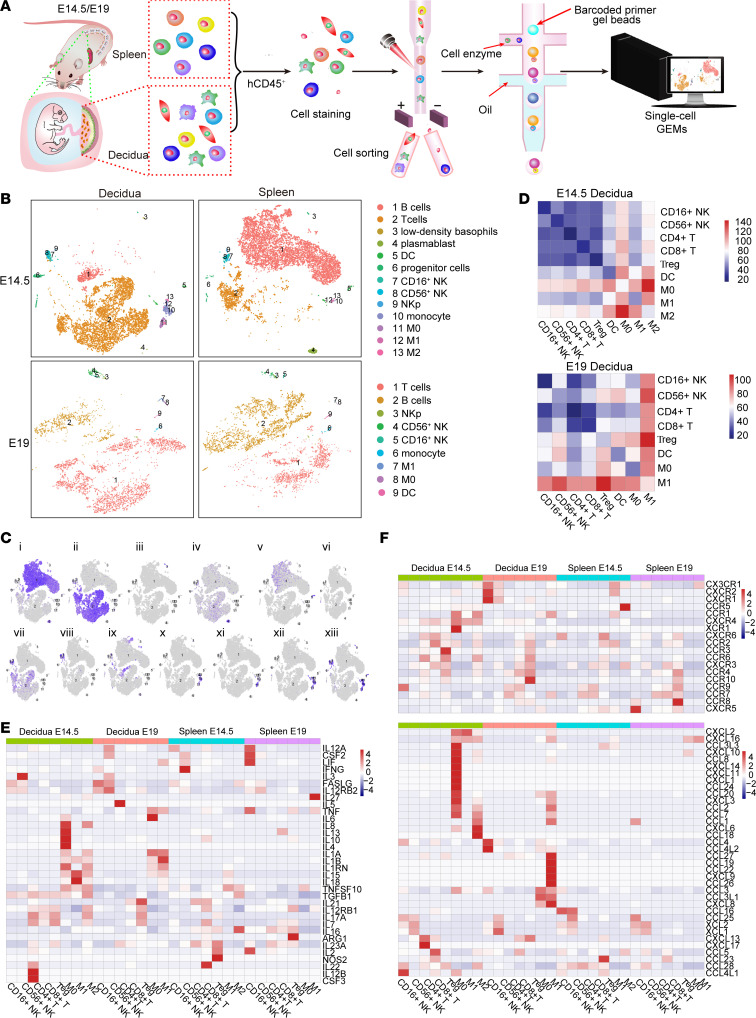
Human immune profile alternation in the decidua and spleen of pregnant HIS mice at E14.5 and E19. Human CD45^+^ cells were purified from the spleen and decidua of pregnant HIS mice made by mating with BALB/c males at mid and late gestation and examined by scRNA-Seq (mother *N* = 1 per time point; E14.5, *n* = 6 decidua, pooled; E19, *n* = 5 decidua, pooled). Each sample represents a single pregnant mouse. Decidua from the same mouse were pooled together. (**A** and **B**) Workflow diagram (**A**) and the t-distributed stochastic neighbor embedding (t-SNE) plots of main cell types (**B**) were shown. GEMs, gel beads in emulsion; DC, dendritic cells; M0, M0 macrophages; M1, M1 macrophages; M2, M2 macrophages; NKp, NK proliferative cells. (**C**) Expression of representative markers for different cell clusters are plotted onto the t-SNE map. Color key from gray to purple indicates relative expression levels from low to high. (i) CD79A (B cells), (ii) CD3D (T cells), (iii) HDC (basophils), (iv) FKBP11 (plasmablasts), (v) LILRA4 (DCs), (vi) SPARC (progenitor cells), (vii) GZMM (CD16^+^ NK), (viii) XCL2 (CD56^+^ NK), (ix) TYMS (NKp), (x) S100A8 (monocytes), (xi) C15orf48 (macrophages), (xii) LYZ (M1), and (xiii) FCER1G (M2). (**D**) The cell-cell interaction profiles at E14.5 decidua and E19 decidua made by CellPhoneDB were shown. (**E** and **F**) Heatmaps show average count of the genes annotated as cytokines (**E**) and chemokine receptors (**F**, upper panel) and chemokine ligands (**F**, lower panel) in different cell types.

Traditionally, materno-fetal immunity was considered under static immunosuppressive status to inhibit maternal versus semiallogeneic fetal immune responses. However, recent studies demonstrated dynamic alternations in the HIS at the materno-fetal interface across the period of pregnancy (3). Human CD45^+^ lymphohematopoietic cells were purified from mouse decidua and spleen at the mid (E14.5) and late (E19) gestation stages and characterized using single-cell RNA-Seq (scRNA-Seq) to examine whether a pregnant HIS mouse model can simulate this process (Figure 3A). A total of 8,217, 9,932, 4,258, and 4,304 qualified single-cell transcriptomes were acquired from E14.5 decidua, E14.5 spleen, E19 decidua, and E19 spleen samples, respectively. We identified 13 cell clusters, including B cells, T cells, basophils, plasmablasts, dendritic cells (DCs), progenitor cells, CD16^+^ NK cells, CD56^+^ NK cells, NKp cells (proliferative NK cells), monocytes, macrophages, M1 macrophages, and M2 macrophages, in E14.5 decidua based on Seurat and annotated with SingleR. Interestingly, identification of the lineage-negative human lymphohematopoietic progenitor subset (cluster 6) at the decidua supports the hypothesis that partial human immune subsets at the decidua might locally differentiate (40). The E19 decidua contained 9 human immune cell clusters, including T cells, B cells, NKp cells, CD56^+^ NK cells, CD16^+^ NK cells, monocytes, M1 macrophages, M0 macrophages, and DCs (Figure 3, B and C, and Supplemental Figure 4). Integration analysis revealed that the composition of human immune cells in the E14.5 decidua from HIS mice was similar to that of human samples collected at similar gestation stage (41), though there were fewer human macrophages, monocytes, and NK cells but more human T and B cells in HIS mice than humans (Supplemental Figure 5). The disappearance of M2 macrophages at the E19 decidua implies a shift in the immune profile from tolerogenic to inflammatory at the materno-fetal interface in this period, as indicated in a previous study (42).

The cell–cell interaction pattern was analyzed using CellPhoneDB (20) to understand the regulation of the human immune profile. Distinct human immune cell–cell interaction patterns (e.g., close crosstalk between macrophages and T cells) were seen between the decidua (Figure 3D) and spleen, which is similar to what was observed in human sample analysis (43) (Supplemental Figure 6). The human M0 and M2 subsets in the E14.5 decidua offered intensive immune-modulating signals toward other lineages of human immune subsets, whereas M1 macrophages were the key node within the cell–cell interaction network at E19 decidua (Figure 3D). The detailed roles of different immune subsets were determined by examining their cytokine and chemokine production pattern. Human decidua M0 macrophages produced a much broader range of cytokines and chemokines compared with other lineages of human immune subsets (Figure 3E). This was consistent with CellPhoneDB data. Interestingly, human decidua macrophages at E14.5 secreted a series of functional immunosuppressive cytokines (such as *IL-4*, *IL-10*, *IL-13*, and *TGFB1*) and angiogenic mediators (such as *IL-8*) that contribute to immune tolerance induction and decidua development. However, they changed to produce a set of inflammatory cytokines (such as *IL-6*, *TNF*, *IL-1A*, and *IL-1B*) (42, 44, 45) at E19, which are crucial for pregnancy termination and fetal delivery. In addition, human decidua macrophages showed potent capabilities for chemokine secretion, wherein the counts of macrophages known for promoting endocytic activity/cell growth and tissue-repairing functions (characterized by mediators such as *CCL18*, *CXCL16*, and *CCL2*) were increased at E14.5, whereas the counts of M0 macrophages known for inflammation induction (characterized by mediators such as *CXCL8* and *CXCL9*) (46) were increased at E19 (Figure 3F). Upregulation of *CXCR4* expression on human E14.5 decidua macrophages suggests that they may be recruited by mouse trophoblast cells that are capable of producing CXCL12 (a CXCR4 ligand) from the periphery (47) (Figure 3F). Decidual Treg (dTreg) cells exhibit different cytokine production patterns between E14.5 and E19; there is a reduction in the expression of *TGFB1* (an immune-inhibitory mediator; ref. 48) and elevation in the expression of *IL21* (a cytokine that negatively regulates Treg cell stability/function; ref. 49). Because TGF-β1 protein undergoes posttranslational modifications to become an active form (50) and no reports showed that Treg cells can produce IL-21 protein (51), we measured TGF-β1 and IL-21 at the protein level by FCM. We found that both IL-21 and TGF-β1 proteins were significantly upregulated in E19 dTreg cells compared with E14.5 dTreg cells (Supplemental Figure 7). These data imply a dynamic transition of the human immune profile from tolerogenic status to inflammatory status at the materno-fetal interface of HIS mice during pregnancy, while the alternation of dTreg cell function in this period requires further examination.

### Decidual Treg cells switch from immune-inhibitory status to activated status from mid to late gestation.

**Figure 4:**
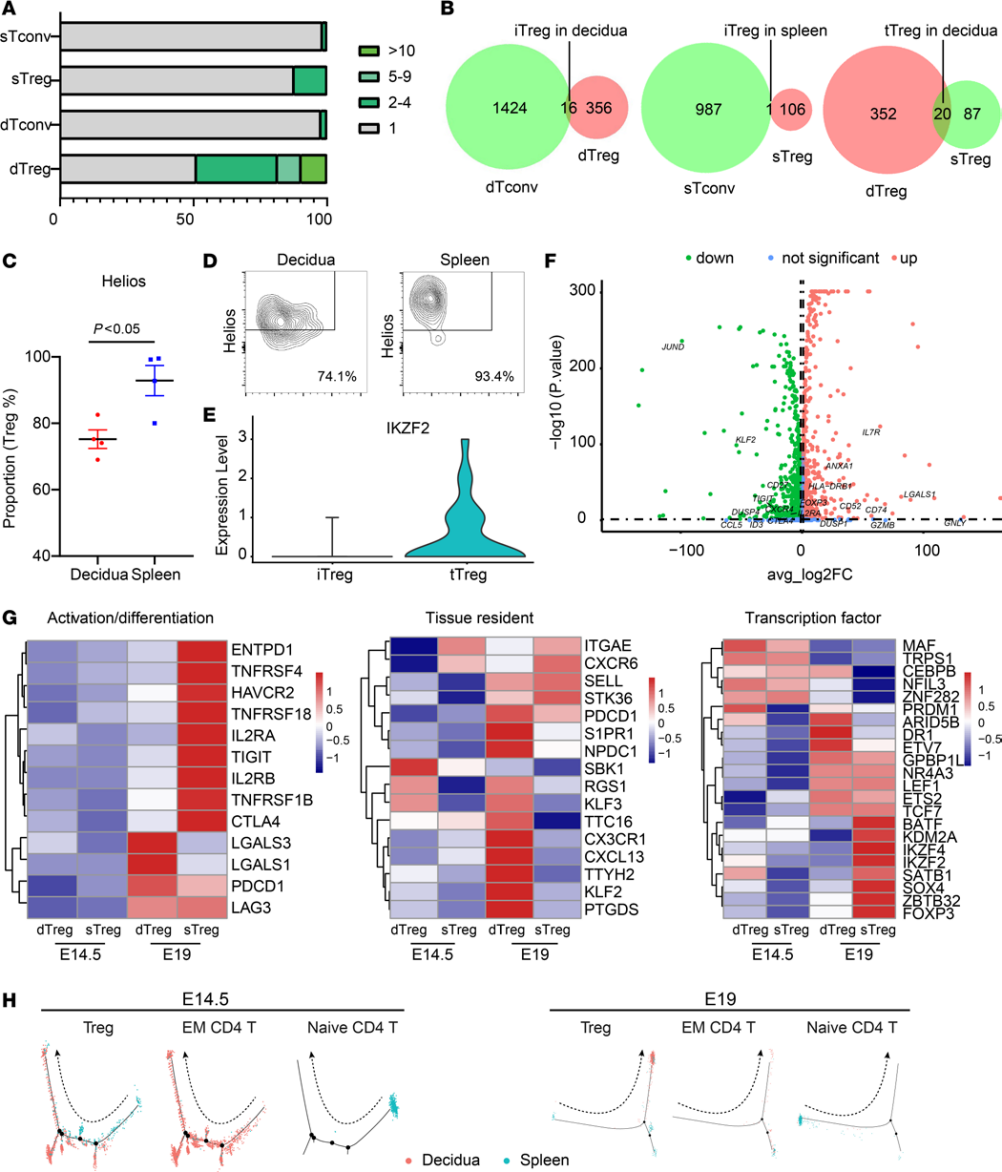
TCR repertoire and immune profile transition of human dTreg cells at E14.5 and E19. (**A**) Quantification of percentage of T cells per clone size. (**B**) Venn diagrams showing the number of TCR clonotypes in dTreg, sTreg, dTconv, and sTconv cells. (**C**) Ratio of Helios^+^ cells in CD4^+^CD25^+^Foxp3^+^ Treg cells in decidua and spleen at E19 (*n* = 4). Significance was determined using paired *t* test. (**D**) Representative flow cytometric profiles of Helios expression in decidual and splenic Treg cells. (**E**) Violin plots showing the expression of *IKZF2* in tTreg and iTreg cells. (**F**) Volcano plot of differential gene expression between dTreg cells at E19 and E14.5. (**G**) Heatmaps show genes related to Treg activation/differentiation (left panel), tissue resident (middle panel), and transcription factors (right panel). (**H**) Pseudotime trajectories of Treg, EM CD4^+^ T, and naive CD4^+^ T cells in decidua and spleen were analyzed using Monocle 2.

Treg cells are an immune subset playing key roles in materno-fetal immune response regulation. Abnormal number or functionality of human Treg cells is clinically associated with several reproductive complications (16, 52). Thus, we focused on characterizing human dTreg cell composition, differentiation, activation, and function alteration in pregnant HIS mice. Initial analysis of the TCR repertoire of Treg cells revealed that around 15% of splenic Treg (sTreg) cells were monoclonal, while approximately 50% of dTreg cells were monoclonal, including approximately 10% of dTreg cells detected with over 10 identical clones. The proportions of monoclonal splenic conventional CD4^+^ T (sTconv) cells and decidua conventional CD4^+^ T (dTconv) cells were much lower (Figure 4A). This finding demonstrated the existence of robust clonal expansion in dTreg cells. TCR overlaps between dTreg and dTconv, sTreg and sTconv, and dTreg and sTreg cells were analyzed. Only 1 out of 107 sTreg cell shared the same TCR with sTconv cells, whereas 16 out of 372 dTreg cells possessed the same TCR as dTconv cells, indicating that increasing numbers of human induced Treg (iTreg) cells were developed at the materno-fetal interface. However, 20 out of 372 dTreg cells shared the same TCR with sTreg cells, suggesting human thymic derived Treg (tTreg) cells that migrated from lymphoid organs also existed at the decidua (Figure 4B). Consistently, *IKZF2* (a classical gene expressed in tTreg cells) was found selectively highly expressed in decidual tTreg cells but not iTreg cells (Figure 4E). Additionally, FCM examination further verified that there were significantly fewer Helios-positive (the protein coded by *IKZF2*) cells within dTreg cells compared with sTreg cells (Figure 4, C and D).

The top 10 genes enriched in dTconv cells, dTreg cells, sTconv cells, and sTreg cells at E14.5 (Supplemental Figure 8A) and E19 (Supplemental Figure 8B) were then clustered. A series of genes that play crucial roles in maintaining Treg cell immune-regulatory function and stability (including *FOXP3*, *TIGIT*, *IKZF2*, *RTKN2*, *ISG20*, *PERP*, *SAT1*, *CXCR4*, *DUSP4*, and *RP11-1399P15*.*1*) were markedly upregulated in E14.5 dTreg cells compared with that in E14.5 sTreg or E14.5 Tconv cells (Supplemental Figure 8A and Supplemental Figure 9A). Specifically, higher expression of *DUSP4* and *RGS1* in E14.5 dTreg cells than in E14.5 sTreg cells may enhance their immunosuppressive function and maintain their retention in the decidua (53, 54). The gene enrichment patterns in E19 dTreg cells were vastly different from that of E19 sTreg cells, wherein a number of genes that are relevant in inflammation, activation, and proliferation (such as *CD52*, *LGALS1*, *S100A4*, *S100A6*, *IL-32*, and *GNLY*) showed a prominent upregulation in E19 dTreg cells (Supplemental Figure 8B and Supplemental Figure 9B). Direct comparison of the gene expression profiles of E14.5 dTreg cells and E19 dTreg cells indicated that the genes involving Treg cell activation and instability (including *HLA-DRB1*, *CD52*, *IL7R*, and *LGALS1*) were upregulated, while the genes critical for Treg cell stability and inhibitory function (including *KLF2*, *TIGIT*, *DUSP4*, and *CD27*) were downregulated (Figure 4F). This finding suggests that a large immune status transition occurred from mid to late gestation. Gene Ontology analysis of differentially expressed genes for E19 dTreg and E14.5 dTreg subsets also revealed that E19 dTreg cells were activated and proliferating (Supplemental Figure 10).

The differences between E14.5 dTreg cells and E19 dTreg cells were analyzed by assessing the differentially expressed genes that are associated with activation/differentiation, tissue location, and transcription factors. E19 dTreg cells upregulated a set of activation/differentiation-related genes (such as *LAG3* and *PDCD1*) and tissue resident–related genes (such as *ITGAE*, *S1PR1*, *CX3CR1*, *CXCL13*, and *KLF2*) while downregulating transcription factor genes known for negatively affecting *FOXP3* stability and immune-inhibitory function (such as *MAF* and *NFIL3*). This suggested that E19 dTreg cells may be actively confronting the inflammatory microenvironment at the decidua right before labor (Figure 4G). E19 sTreg cells also showed upregulation of a series of genes associated with immune regulation (such as *IL2RA*, *TIGIT*, and *CTLA4*) and transcription factors (such as *FOXP3* and *IKZF2*) that are essential for enhancing Treg cell immune-inhibitory function, which differed from that of E19 dTreg cells. This might suppress potential autoimmune disorders in the whole body driven by the immune reactions during fetal delivery. Temporal dynamic trajectory analysis was performed by Monocle 2 assays (55) to better understand the differentiation status of dTreg cells. Naive T cells were found at the start point of the trajectory and gradually transitioned toward effector memory (EM) T cells, which was consistent with previous reports (56). E14.5 dTreg cells and E14.5 sTreg cells exhibited a transitional distribution in the trajectory, with the differentiation of E14.5 sTreg cells being closer to the start point than that of E14.5 dTreg cells. E19 dTreg cells and E19 sTreg cells were concentrated in different terminally differentiated regions (Figure 4H). These data revealed an immunological feature transition of human dTreg cells in pregnant HIS mice during pregnancy.

### Human Treg cells suppress maternal versus fetal T cell reactions and maintain immune tolerance.

**Figure 5:**
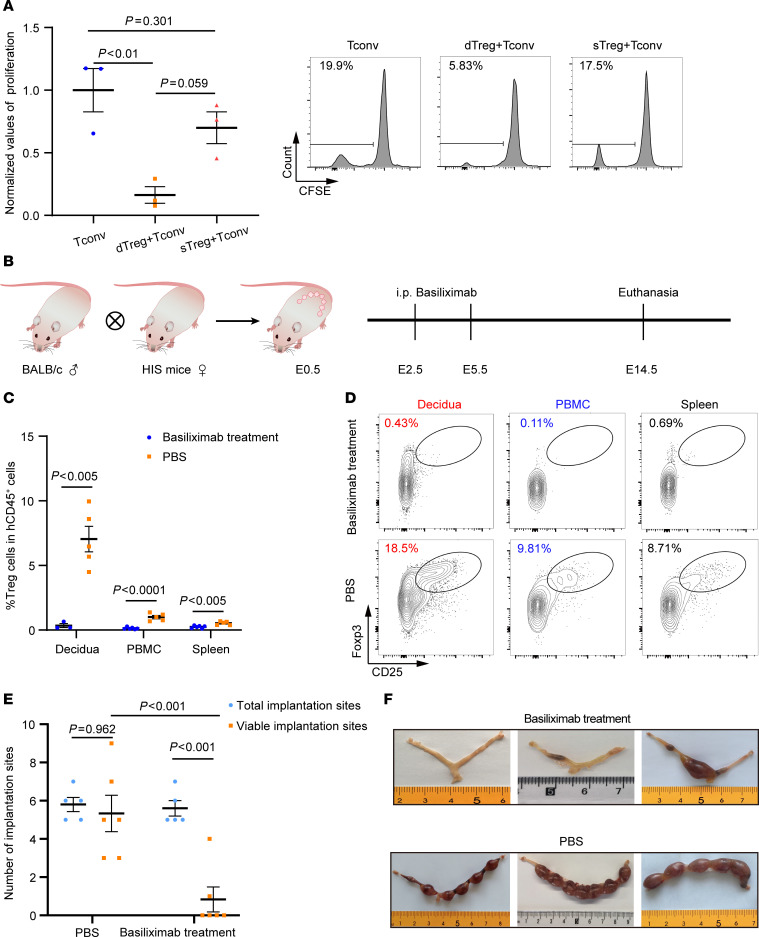
Inhibition of human Treg cells causes pregnancy termination and fetal rejection in pregnant HIS mice. Treg cell inhibitory function examination. (**A**) Summarized data (*n* = 3; mean ± SEM) of the immune-inhibitory effect of human dTreg or sTreg cells on human sTconv cell proliferation in the presence of 30 Gy–irradiated BALB/c splenic cells. Representative FCM profiles of Tconv cell proliferation. Treg/Tconv = 1:16, BALB/c stimulator cells/Tconv = 1:2. Statistical differences were determined with 1-way ANOVA for multiple-variable comparisons. (**B**) Schematic outline of the experimental design. Anti-CD25 mAb (basiliximab; *n* = 6) or equal volume of PBS (*n* = 5) was i.p. injected into pregnant HIS mice (200 μg/mouse) made by mating with BALB/c males at E2.5 and E5.5, and the mice were euthanized at E14.5. Percentages (mean ± SEM, **C**) and representative FCM profiles (**D**) of Treg cells after anti-CD25 mAb injection were shown. Statistical analyses were performed using unpaired *t* test. (**E**) The number of implantation sites of pregnant HIS mice treated with PBS (*n* = 5) or basiliximab (*n* = 6). Statistical differences were calculated with 2-way ANOVA. (**F**) Representative photos of the uteri harvested from HIS mice treated with basiliximab (upper panel) or PBS (lower panel).

Last, we investigated whether the human materno-fetal immune system in HIS mice was functional. Human decidua and splenic CD4^+^CD25^+^ Treg cells were purified from pregnant HIS mice and incubated with the CFSE-labeled human CD45^+^CD25^–^ splenic cells (responder cells from pregnant HIS mice) in the presence of 30 Gy irradiated BALB/c mouse splenic cells (stimulator). The proliferation of responder cells was quantified 4 days later using FCM. Proliferation of maternal human splenic CD4^+^ effector T cells after stimulation with paternally (BALB/c) derived antigen reflects the presence of fetal reactive human maternal T cells (Figure 5A). STreg cells were capable of suppressing Tconv cells at 1:2 ratio (Treg/Tconv) (Supplemental Figure 11). However, under a low Treg/Tconv ratio (1:16) condition, the maternal versus fetal T cell reactions (MvFRs) of maternal T cells were significantly inhibited by dTreg but not by sTreg cells (or were only minimally suppressed; Figure 5A).

**Figure 6:**
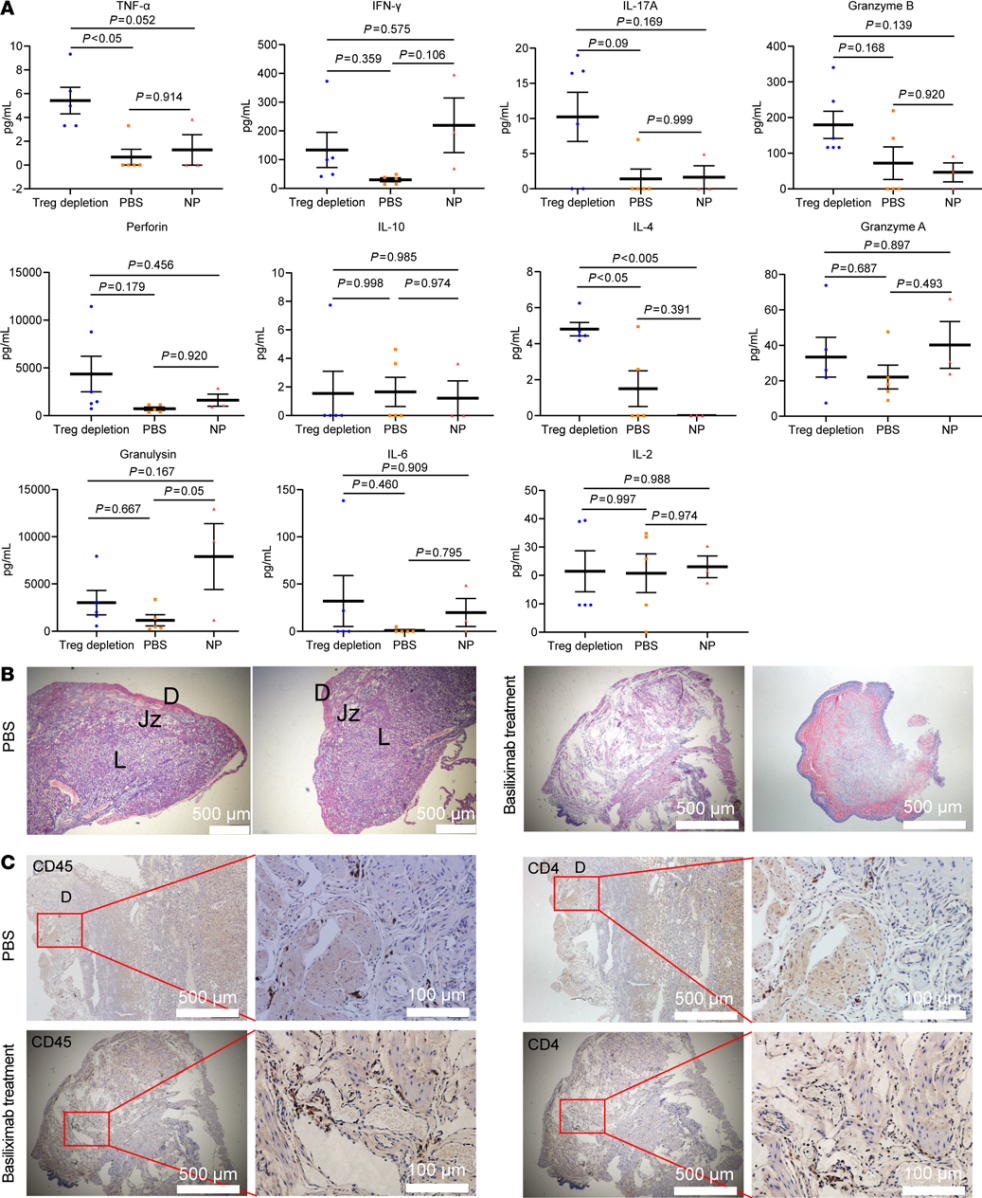
Inhibition of human Treg cells causes an inflammatory response in pregnant HIS mice. (**A**) Concentration (mean ± SEM) of human TNF- α, IFN-γ, IL-17A, granzyme B, perforin, IL-10, IL-4, granzyme A, granulysin, IL-6, and IL-2 in mouse serum. Statistical differences were determined with 1-way ANOVA for multiple-variable comparisons. NP, nonpregnant HIS mice. (**B**) Representative H&E images of the decidua in PBS group (left) and the resorbed embryos collected from the basiliximab treatment group (right). D, decidua; Jz, junctional zone; L, labyrinth zone. (**C**) IHC examination of the placenta (PBS) or uterus (basiliximab treatment) section stained with anti-human CD45 and CD4 antibodies. Scale bars for each picture are shown at lower right corner.

We then evaluated the roles of human Treg cells in maintaining immune tolerance in vivo during pregnancy. Basiliximab is a monoclonal antibody of the IgG1κ subtype that specifically targets CD25 (the α subunit of the IL-2 receptor) and can inhibit the interaction of IL-2 with Treg cells, causing IL-2 starving (57). When administered to humans, basiliximab causes a significant reduction in the number of circulating Treg cells (58, 59). Using a conventional pregnant mouse model, it has been shown that Treg cells are necessary for maintaining materno-fetal immune tolerance during early stages, but not late stages, of pregnancy (60, 61). Pregnant HIS mice were treated at E2.5 and E5.5 with pretitrated basiliximab (Supplemental Figure 12) or with the same volume of PBS as controls and euthanized at E14.5 for examination (Figure 5B). The number of human CD25^+^Foxp3^+^CD4^+^ Treg cells was markedly reduced in the PBMCs, spleen, and decidua (Figure 5, C and D). Severe embryo resorption was observed in basiliximab-treated mice but not in control mice (Figure 5, E and F). Moreover, markedly elevated levels of inflammatory mediators (such as TNF-α, IFN-γ, IL-17A, granzyme B, perforin, and IL-4), but not antiinflammatory molecules (such as IL-10), were detected in the sera of basiliximab-treated mice, but not control mice (Figure 6A). The majority of these inflammatory mediators (except IFN-γ) did not increase in nonpregnant HIS mice after basiliximab treatment (Figure 6A). This finding indicated that elevation of these inflammatory cytokines is attributed to materno-fetal immunological rejection. Moreover, histological examination of the embryonic resorption site demonstrated severe tissue damage and human T cell infiltration in the embryo of basiliximab-treated mice but not in control mice (Figure 6, B and C). These results verified that the human materno-fetal interface immunity in pregnant HIS mice was functional and capable of developing MvFRs and rejecting fetuses after inhibition of Treg cell activity.

## Discussion

The development of humanized mouse models capable of representing human materno-fetal immunity will facilitate the investigation of human reproductive immunity and related drug discovery. Herein, we constructed a pregnant HIS model with a multilineage human immune subset reconstitution at the materno-fetal interface, which had a specialized composition, phenotype, and transcription pattern compared with that in lymphoid organs. Human materno-fetal immune profiles switch from a tolerogenic status to an inflammatory status between mid and late gestation in pregnant HIS mice. Importantly, the human materno-fetal immunity reconstructed in mice is functional, and disruption of immune tolerance through Treg inhibition leads to fetal rejection attributed to robust MvFRs.

Immune components at the materno-fetal interface are notably different from those found in the peripheral blood and lymphoid organs and accomplish their special mission during pregnancy. Previous reports have demonstrated that NK cells identified with a tissue residential phenotype are highly enriched at the materno-fetal interface and play crucial roles in immune surveillance and placenta development (13). However, how these human NK cells develop remains unknown owing to the lack of in vivo platforms. Mouse tissue-resident NK (trNK) cells may be derived from local lymphohematopoietic progenitor cells (62). Herein, we revealed that, in pregnant mice whose HIS was reconstructed at the adult stage, the counts of human CD56^+^CD16^–/lo^ NK cells (known for their cytokine production potential) were significantly higher at the decidua than in the PBMCs or spleen. Furthermore, there were significantly higher ratios of CD49a^+^ and CD49a^+^CD103^+^ trNK cells in the decidua than in the PBMCs and spleen. These data demonstrate that human trNK cells at the materno-fetal interface can be developed from the adult HIS. Elevated levels of human decidual trNK cells were found in pregnant HIS mice made by mating with allogeneic BALB/c males compared with those in HIS mice that mated with syngeneic NSG males, implying that immune stimulation from fetal antigens may contribute to human decidual trNK cell development. Interestingly, human lymphohematopoietic progenitor cells (cluster 6 in Figure 3B) were detected in the decidua of pregnant HIS mice, which supports the possibility that human decidual trNK cells are also locally differentiated.

Macrophages are specialized phagocytic cells that engulf dying trophoblast cells at the materno-fetal interface to prevent exposure to the fetal antigens that could trigger maternal immune reactions (63). Moreover, these macrophages secrete immunomodulatory cytokines, chemokines, or tissue growth factors to regulate immune functions and placenta development (46). Herein, we revealed that the levels of CD14^+^HLA-DR^+^ human macrophages were significantly higher in the decidua than in the PBMCs or spleen at E14.5 in pregnant HIS mice; these cells exhibited the CD206^+^ M2 phenotype but not the CD80^+^ M1 phenotype. This suggests that they have an immune-inhibitory function, which is consistent with the results of clinical sample examinations (64). Notably, we found that human decidua macrophages are the key node in the immune cell–cell interaction network based on a CellPhoneDB analysis of scRNA-Seq data. The cytokines secreted by the human decidual macrophages (TGF-β and IL-10) may contribute to the stability and differentiation of human dTreg cells; production of human IL-15 may facilitate the local survival and differentiation of human dNK cells. The expression of chemokine receptors (including CXCR4, CXCR6, CCR1, and CCR2) on Treg and NK immune subsets contributes to their recruitment to the decidua from the periphery (47, 65, 66). These data indicate that the pregnant HIS model can precisely represent the characteristics of human decidual macrophages in shaping the immune atmosphere at the materno-fetal interface.

The traditional conceptual model theorized that reproductive immunity is under a static immunosuppressive status to ensure maternal immune tolerance to the MHC-incompatible fetus. However, recent studies have revealed that the status of reproductive immunity experiences “pro-inflammatory,” “antiinflammatory,” and “pro-inflammatory” changes at the early, mid, and late gestational stages, respectively, to coordinate pregnancy events such as embryo implantation, fetus growth, and pup delivery (67). Human immune profiles at the decidua in pregnant HIS mice were similar to those found in human beings: there was a vast alteration between the mid and late gestational stages (3, 42, 68–70). This was observed by the disappearance of M2 macrophages and reduced production of antiinflammatory cytokines (such as IL-10 and TGF-β). Meanwhile, functions of pro-inflammatory cytokines (including IL-1A, IL-2B, and IL-6) increased during this period. Specific upregulation of IL-6 by human decidual macrophages at E19 may contribute to successful fetal delivery (42). In addition, dTreg cells exhibit intensive immune profile alterations from a canonical immunosuppressive feature to the activated and terminal differentiated status during this process. Human immune profiles in the lymphoid organs of the pregnant HIS mice showed different alternation trends in this period. For instance, E19 sTreg cells showed a marked upregulation of the genes associated with immune-inhibitory functions (such as *FOXP3* and *IKZF2*, *TIGIT*, *CTLA4*, and *IL2RA*) compared with E14 sTreg cells, which may contribute to suppressing potential inflammation in the whole body, which occurs during fetal delivery. These data suggest that the pregnant HIS mouse model may serve as a unique tool to study the impact and underlying mechanism of dynamic human immune profile alternations at the materno-fetal interface in vivo.

Exposure of the paternally derived allogeneic antigen to the maternal immune system drives MvFRs. The specialized immune atmosphere at the materno-fetal interface ensures effective control of potential MvFRs as well as the maintenance of immune surveillance to pathogens (3). Herein, we found that human splenic T cells in pregnant HIS mice can significantly proliferate in the presence of paternal cells; this indicates the existence of functional MvFR human T cells in the maternal immune system. Moreover, inhibition of the human Treg cells using an anti-CD25 antibody injection terminated the pregnancy process and caused fetal resorption in pregnant HIS mice. Markedly elevated levels of a series of inflammatory cytokines were detected in the Treg-depleted pregnant humanized mouse sera compared with those in the control pregnant mice or Treg-depleted nonpregnant HIS mice. These data demonstrate that the human materno-fetal immune system in pregnant HIS mice is functional and can mediate robust antifetus immune responses after immune tolerance is broken.

Successful pregnancy requires effective induction of maternal immune tolerance to the fetal antigens wherein Treg cells play crucial roles (16). Abnormal Treg cell number and functionality are associated with a series of reproductive disorders, including recurrent spontaneous abortion and intrauterine growth restriction (71, 72). In general, Treg cells are composed of tTreg and iTreg cells (73). A lack of either cell type or function deficiency may cause immune disorders (74, 75). A fetus-derived antigen may drive the differentiation of mouse iTreg cells at the materno-fetal interface, though whether a human counterpart exists remains unknown (76). Herein, we identified human iTreg cells that share the same TCR as Tconv cells at the materno-fetal interface in pregnant HIS mice. These iTreg cells were deficient in *IKZF2*, the gene encoding the Helios protein, which contributes to the stability of the Foxp3 protein and ensures tTreg cell function (77). These data validate the hypothesis that human iTreg cells can be converted from Tconv cells at the materno-fetal interface. Decidual Treg cells have been shown to possess a stronger capability to suppress MvFRs than Treg cells in the periphery, based on the clinical sample examination (78). Consistently, we found that dTreg cells can suppress MvFRs more effectively than sTreg cells in pregnant HIS mice. These data demonstrate that this pregnant HIS model may serve as a useful tool to study the fate and function of human Treg cells during pregnancy.

The proportions of human NK cells and myeloid cells at the materno-fetal interface in HIS mice are significantly lower than those in humans and normal mice; conversely, a higher number of human T and B cells are present at the materno-fetal interface in HIS mice compared with both humans and normal mice (79–81). Thus, the current pregnant HIS model still has some deficits that need to be corrected to represent human materno-fetal immunity more precisely. First, NK cells make up the largest cell population at the human decidua, whose ratios reach 30%–70% among all the immune cells (4). However, its ratios reach only around 4% in the decidua of pregnant HIS mice. Mouse IL-15 does not cross-react with human cells (82) owing to the low similarity of amino acid sequences between mice and humans. Extravillous trophoblast cell–derived IL-15 plays crucial roles in promoting NK cell differentiation and survival at the decidua (83). Similarly, human myeloid cells were also not well developed in the HIS mice because of the poor cross-reaction of some mouse cytokines, such as GM-CSF with IL-3 and M-CSF (84). Therefore, the application of immunodeficient mice as recipients that secrete corresponding human cytokines in an appropriate manner (85) may further improve this model to study the roles of human dNK cells and myeloid cells in pregnancy or relevant reproductive diseases. Second, MvFRs in pregnant HIS mice are caused by human T cell reactions to the alloantigens presented on mouse MHC molecules that are different from those in humans, wherein they are induced by HLA molecule incompatibility. Thus, the usage of HLA-knockin immunodeficient mice (86) as recipients for HIS model generation would further elevate its value for human T cell function research during pregnancy.

In summary, we developed a humanized mouse model with specialized and functional multilineage human lymphohematopoietic cell reconstitution at the materno-fetal interface. Although there are notable differences between human and rodent pregnancies, such as gestation period, placental anatomy, incidence of multifetal pregnancies, and the arrival and functionality of fetal adaptive immune cells after birth (39, 87, 88), the human materno-fetal immunity developed in the HIS mouse model showed many similarities to that in humans. This model may serve as a powerful in vivo platform for basic human reproductive immunity investigations and provides a tool to develop and test drugs or therapeutic interventions for human reproductive immune disorders. This model may also help address some interesting concerns in this field, including how the early HIS develops and functions at the materno-fetal interface, which is still not well understood (89), and how human materno-fetal microchimerisms form (90) and influence both maternal health and the offspring’s well-being. Spatial transcriptomics offers a potent tool for comprehensively understanding physiological processes during early pregnancy, including decidualization and trophoblast differentiation (19, 89). Employing spatial transcriptomics technology in this HIS pregnant mouse model could further enhance our understanding of human materno-fetal interface immunity.

## Methods

### Sex as a biological variable.

Female NSG (NOD/SCID Il2rg^–/–^, also named NCG) mice were used for establishing HIS mouse models, and male NSG or BALB/c mice were used for mating with HIS mice to study the development of human materno-fetal interface immunity.

### Animals and human samples.

The NSG and BALB/c mice were purchased from GemPharmatech Co., Ltd., and Beijing Vital River Laboratory Animal Technology Co., Ltd, respectively, and housed in a specific pathogen–free animal facility. All the mice used were between 6 and 16 weeks of age. Discarded human fetal samples of gestational ages 17–21 weeks were obtained from The First Hospital of Jilin University with informed consent and prepared as reported previously (32).

### Preparation of HIS mice.

Female NSG mice at approximately 6 weeks of age were preconditioned with 1.75 Gy TBI or limb local irradiation (main body covered by a 6.5 mm–thick lead box with the 4 limbs exposed) using hard x-rays (Rad Source RS2000 Pro Biological Irradiator), or i.p. injections with busulfan (20 mg/kg), and then implanted with 1 mm^3^ human fetal thymic tissues under their renal capsule together with a human CD34^+^ fetal liver cells (around 2 × 10^5^) intravenous injection as shown in our previous study (33). Male NSG or BALB/c mice were randomly introduced to the pregnant HIS mice at 7–12 weeks after transplantation, and successful pregnancies were confirmed through the detection of a vaginal plug, which was designated as E0.5.

### Treg depletion in pregnant humanized mice.

To deplete the human Treg cells, anti-CD25 monoclonal antibodies (200 μg/mouse, basiliximab, Novartis AG), or sample volumes of PBS as a control, were i.p. injected into pregnant HIS mice at E2.5 and E5.5. The mice were euthanized by cervical dislocation at E14.5 for further analysis.

### Human lymphohematopoietic cell preparation.

Fresh mouse decidual samples containing some placenta tissues (no human immune cells) were separated. After being minced into small pieces with scissors, they were washed with PBS and digested in a medium containing 0.1% collagenase type IV (MilliporeSigma) and 0.01% DNase I (MilliporeSigma) for 30 minutes at 37°C in a water bath shaker. The cell suspension was filtered through a 40 μm cell strainer (Falcon) and centrifuged at 500*g* for 5 minutes. The pellets were lysed with an RBC lysis buffer (Solarbio) for 1 minute at room temperature and washed before use. The mouse spleen tissue was passed through a 70 μm cell strainer (Falcon); RBCs were lysed and washed using PBS. The blood samples were carefully laid onto Lymphoprep (Axis-shield) and centrifuged at 800*g* for 30 minutes without brakes; the mononuclear cells were harvested from the interface and washed in PBS for use.

### Flow cytometric analysis.

After being stained with fluorochrome-conjugated monoclonal antibodies as listed in Supplemental Table 1 and examined via FACSFortessa (BD Biosciences), multicolor FCM analysis was used to determine the constitution and phenotype of human immune cells in the humanized mice. All the decidua from the same host were pooled together for analysis. A Transcription Factor Staining Buffer Set (BD Biosciences) was utilized to stain the Foxp3 intracellularly. In the analysis process, dead cells were excluded by gating out propidium iodide–positive (extracellular staining) or Aqua-positive (intracellular staining) and lower forward scatter cells. FCS files were analyzed using FlowJo v10.0.7 (Tree Star Inc.).

### Histology.

The decidual tissue together with some junctional zone and labyrinth zone tissues of the pregnant mice were treated with 4% paraformaldehyde for 24 hours and embedded in paraffin. Thereafter, 4 μm sections were prepared for H&E and IHC staining. Briefly, after antigen retrieval, the sections were stained with anti-human CD45 or CD4 primary antibodies (Supplemental Table 2) for 1 hour, followed by incubation with peroxidase-labeled secondary antibodies (1:100; GE Healthcare, now Cytiva) and a DAB kit (MXB biotechnologies). The slides were imaged under a microscope (Olympus IX51, Tokyo). To determine the ratio of junctional zone/labyrinth zone, we derived the area of the junctional zone and labyrinth zone stained with H&E.

### ScRNA-Seq.

The single-cell sequencing technology adopts microfluidic technology to wrap a single cell in an oil droplet containing magnetic beads and realize the traceability of the source nucleic acid of each cell through the single-cell barcode on the magnetic beads. Live human CD45^+^ cells were purified from the lymphohematopoietic cell suspension of the decidua and spleen using a BD Influx cell sorter (BD Biosciences) and collected in PBS containing 0.04% of BSA. The cell numbers were immediately counted with a LUNA-FL Dual Fluorescence Cell Counter (Logos biosystems) and loaded on to the 10x Genomics Chromium system. The 10x Genomics v2 libraries were prepared according to the manufacturer’s instructions. The samples were processed to generate a library with a unique barcode for the following sequencing separately. Single-cell 5′ and V(D)J sequencing libraries were constructed with a Chromium Single Cell 5′ Library & Gel Bead Kit plus Chromium Single Cell V(D)J Enrichment Kit (Human T Cell, PN-1000005), following the 10x Genomics protocol. The qualified library was sequenced on an Illumina HiSeq 4000 platform (paired-end; read 1: 26 cycles; i7 index: 8 cycles, i5 index: 0 cycles; read 2: 98 cycles). The data were preprocessed with Cell Ranger (10x Genomics, version 3.1.0) and the Seurat package (Release 3.2.2). Clusters were annotated by the SingleR package (Release 1.2.4). A detailed description of the procedure is in the Supplemental Methods.

### Immune-inhibitory function assay of Treg cells.

The CD4^+^CD25^+^ Treg cells and non-Treg cells (other than the CD4^+^ CD25^+^ T cells) were sorted from the cell suspension of the decidua and spleen of the pregnant HIS mice made by mating with the BALB/c males. Non-Treg cells were labeled with 1 mM carboxyfluorescein succinimidyl ester (CFSE; Life Technologies, Thermo Fisher Scientific) and incubated with 30 Gy–irradiated BALB/c spleen cells with or without decidua Treg cells or spleen Treg cells at ratios of 1:16 (Treg cells versus non-Treg cells). Four days later, the proliferation of human T cells was determined by the dilution of CFSE dye under a BD Biosciences FACSCanto II cytometer.

### Quantification of cytokine production.

Serum was separated from the peripheral blood of the humanized mice after centrifugation at 1,000*g* for 10 minutes and stored at –80°C. Legendplex multianalyte flow assay kits (BioLegend) containing IL-2, IL-4, IL-6, IL-10, IL-17A, IFN-γ, TNF-α, soluble Fas, soluble FasL, granzyme A, granzyme B, perforin, and granulysin were used according to the manufacturer’s instructions. The samples were examined on a FACSCanto II flow cytometer (Becton Dickinson) and analyzed using the Legendplex Data Analysis V8.0 software (BioLegend).

### Statistics.

Paired 2-tailed *t* tests or unpaired 2-tailed *t* tests were used to determine the statistical significance between 2 groups. Statistical differences were determined with 1-way ANOVA or 2-way ANOVA for multiple-variable comparisons. All statistical analyses were performed using the GraphPad Prism 8 software. The FCM data were analyzed with FlowJo V10. All the quantitative data are expressed as the mean ± SEM. *P* < 0.05 was considered statistically significant.

### Study approval.

The Institutional Review Board and Institutional Animal Care and Use Committee of The First Hospital of Jilin University approved the protocols for the use of human tissues and animals. Discarded human fetal samples were obtained with written informed consent.

### Data availability.

All data needed to evaluate the conclusions in the paper are present in the paper or the supplement. Values for all data points in graphs are reported in the Supporting Data Values file. The data that support the findings of this study are openly available in the National Center for Biotechnology Information Gene Expression Omnibus, reference number PRJNA913700.

## Author contributions

ZH and SD conceived the study. SD, MX, YL, JZ, and HMT developed methodology. CS, YZM, PX, and YHS performed sample acquisition. CF and SD performed scRNA-Seq data analysis. JH, YGY, and ZH supervised. SD, CS, YGY, and ZH acquired funding. SD and ZH wrote the original draft. ZH, YGY, and JH reviewed and edited the manuscript.

## Supplementary Material

Supplemental data

Supporting data values

## Version 1 10/22/2024

Electronic publication
