# Supplementary material for: Development of a humanized mouse model with functional human materno-fetal interface immunity
Source: JCI Insight. 2024 Oct 22;9(20):e176527. doi: 10.1172/jci.insight.176527 (PMC11529984; doi:10.1172/jci.insight.176527)
Supplement: Supplemental data [file jciinsight-9-176527-s192.pdf]

Supplementary Information

Development of a humanized mouse model with functional human maternal–fetal interface immunity

Shuai Dong[1,2], Cong Fu[1,2], Chang Shu[1], Min Xie[1,2], Yan Li[1,2], Jun Zou[1,2], Yi-Zi Meng[1,2], Peng Xu[1,2], Yan-Hong Shan[1], Hui-Min Tian[1,2], Jin He[1*], Yong-Guang Yang[1,2,3*], Zheng Hu[1,2*]

[1]Key Laboratory of Organ Regeneration & Transplantation of Ministry of Education, Department of obstetrics, Obstetrics and Gynaecology Center, The First Hospital of Jilin University, China

[2]National-Local Joint Engineering Laboratory of Animal Models for Human Diseases, China

[3]International Center of Future Science, Jilin University, China

* **Corresponding author:** Zheng Hu, Yong-Guang Yang, Jin He

Address correspondence to:

Zheng Hu, National-Local Joint Engineering Laboratory of Animal Models for Human Diseases, 1977 Xinzhu Rd, Changchun, Jilin, P.R. China 130062. Tel (office) +86-431-80539007. Email: zhenghu@jlu.edu.cn.

Yong-Guang Yang, National-Local Joint Engineering Laboratory of Animal Models for Human Diseases, 1977 Xinzhu Rd, Changchun, Jilin, P.R. China 130062. Tel (office) +86-431-80539001. Email: yongg@jlu.edu.cn.

Jin He: Department of obstetrics, The First Hospital of Jilin University, 71 Xinmin Street, Changchun, Jilin, P.R. China 130021. Tel (office) +86-431-88785201. Email: hejin@jlu.edu.cn.

Conflict-of-interest statement: The authors have declared that no conflict of interest exists.

MATERIALS AND METHODS

Data preprocessing with Cell Ranger and Seurat

Single-cell sequencing data were aligned and quantified using the Cell Ranger Single-Cell Software Suite against the hg19 human reference genome. Quality of cells were then assessed based on three metrics: (1) The number of total UMI counts per cell (library size) were < 40000; (2) The number of detected genes per cell were < 5000 and > 500; (3) The proportion of mitochondrial gene counts were $< 20\%$. Downstream analyses, such as normalization, clustering, differential expression analysis and visualization, were performed using “Seurat” package in R software (version 4.0.2). Cell cycle effect was considered, scored, evaluated and eliminated during normalization. Clusters were identified using the community identification algorithm as implemented in the Seurat ‘FindClusters’ function, and the resolution parameter was tuned to produce a proper number of clusters and capture most of the biological variability. tSNE analysis was performed using the RunTSNE function with default parameters. Differential expression analysis was performed based on the Wilcoxon rank-sum test.

Cluster specific gene identification and marker-based classification

Based on the clusters produced, the Seurat ‘Find All Markers’ function was used to identify cluster-specific marker genes with thresholds p value < 0.05. Clusters were annotated by SingleR package, and results of 3 reference databases comparisons (Nover shtern Hematopoietic Data (1), Monaco Immune Data (2), Database Immune Cell Expression Data (3) were combined. The first round of

clustering identified six major cell types including T and NK cells, monocyte, B cells, DC, basophils, plasmablast and progenitor cells. A second round of clustering was performed on T, NK and monocyte cells to subdivide.

Enrichment analysis

To determine transcriptional signatures, R package "ClusterProfiler" (Release 3.16.1) (4) was used for functional enrichment analysis of DEGs in different cell types, and GO biological processes at the significant level (q-value < 0.05) were employed. For heatmap representation, the Seurat 'DoHeatmap' function was used.

Cell–cell communication analysis

CellPhoneDB v.2.0 (5) were used to infer cell-cell communication by combining expression of multi-subunit receptor-ligands complexes, default parameters were used. Cell-cell interactions heatmaps were generated using pheatmap R package.

Single-cell pseudotime trajectory analysis

Pseudotime cell developmental trajectory analysis was analyzed by Monocle 2 package (v2.16.0) to explore the cell-state transitions. To analysis of cell-cell communication by interactions, we use CellPhoneDB algorithm (www.CellPhoneDB.org) (p < 0.05).

Single-cell TCR analysis

TCR sequences were aligned to the GRCh38 reference genome using the 10× Genomics Cell Ranger V(D)J pipeline. Only single-cell barcodes with one or two TCR α chains and one TCR β chain sequences and for which the transcriptome was available were kept for further analysis. Clonotypes were assigned to cells based on unique paired V-gene/CDR3/J-gene of TRA-TRB sequences.

Integration analysis with published human data

Single-cell sequencing data on the human cell composition in the decidua of E14.5 HIS mice were compared with data from their human counterparts collected at 20-34 week (https://doi.org/10.6084/m9.figshare.23264102.v1.) through integration analysis.

Supplementary figure legends

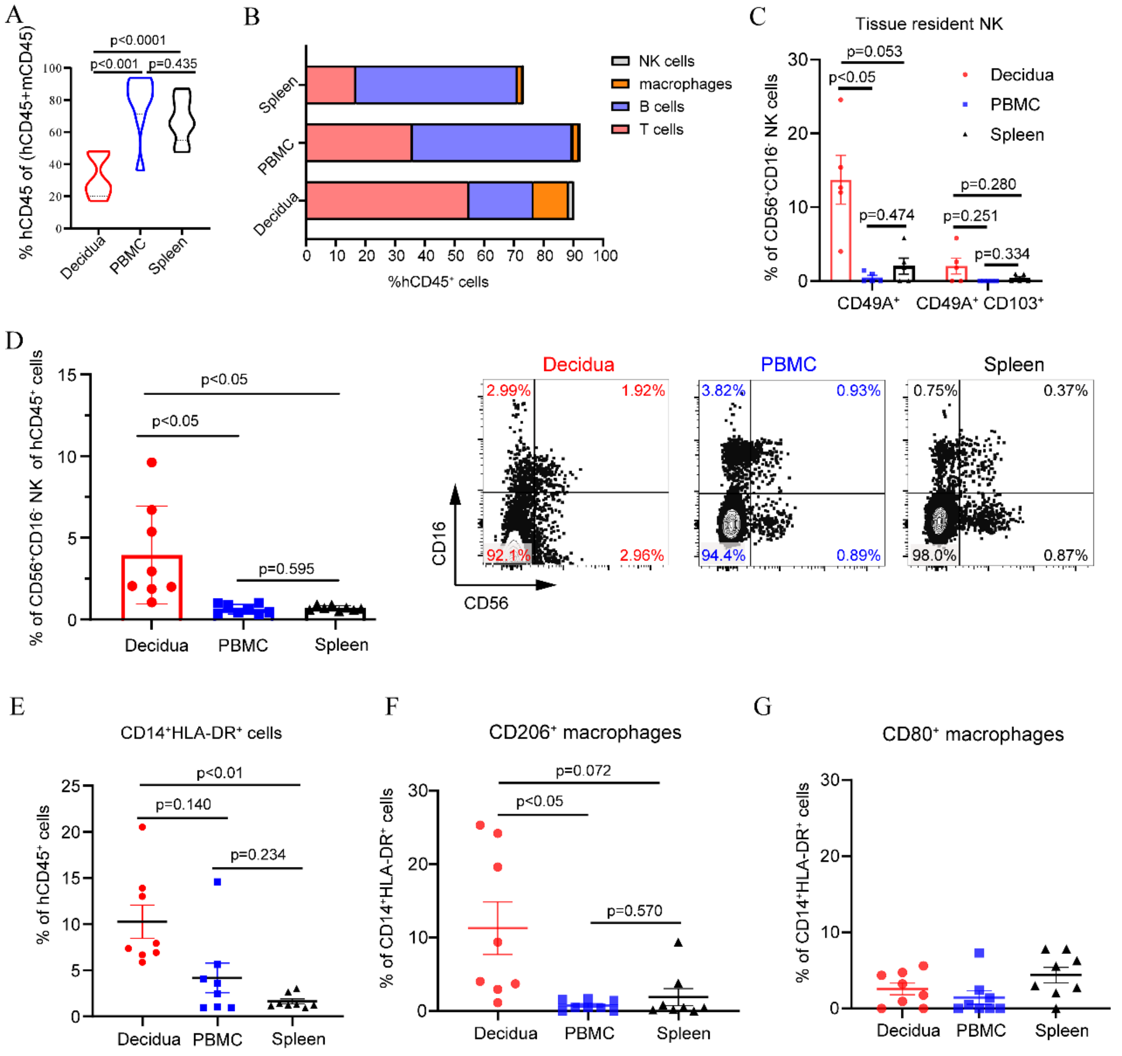

Figure S1. Human immune cell reconstitution in pregnant HIS mice made by mating with NSG males at E14.5. (**A**-**B**) Shown are human lymphohematopoietic chimerism (A; mean ± SEM) and ratios (B; mean ± SEM) of specific immune subsets within human CD45$^+$ cells in MFI, PBMC and spleen of HIS mice (n = 8). (**C**) Percentages (mean ± SEM) of CD56$^+$CD49a$^+$ and CD56$^+$CD49a$^+$CD103$^+$ cells within CD56$^+$CD16$^-$ NK cells. (**D**) Percentages (left; mean ± SEM) and representative FCM profiles (right) of CD56$^+$CD16$^-$ NK cells. (**E**-**G**) Percentages (mean ± SEM) of total human macrophages (E), CD206$^+$ M2 (F) and CD80$^+$ M1 (G) macrophages. Statistical differences were determined with one-way ANOVA for multiple variable comparisons.

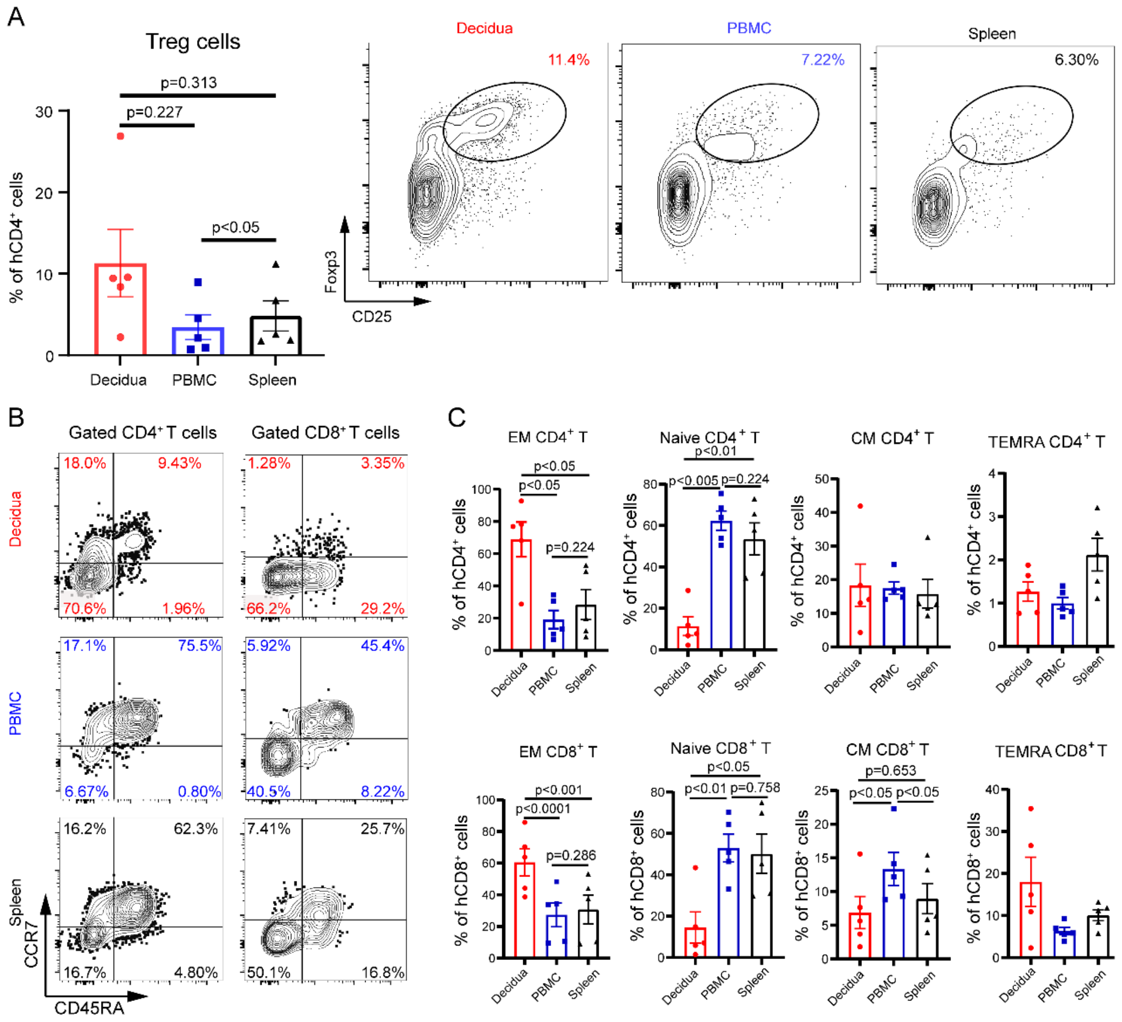

Figure S2. Characterization of human T cell compartment in pregnant HIS mice that mated with NSG males at E14.5. (**A**) Percentages (n =5; mean ± SEM) of human Treg cells within CD4$^+$ T cells. (**B**) Representative FCM profiles of CD45RA and CCR7 expression in human CD4$^+$ and CD8$^+$ T cells. (**C**) Summarized results (mean ± SEM; n = 5) of the percentages of EM T cell (CD45RA$^-$CCR7$^-$), Naive T cell (CD45RA$^+$CCR7$^+$), TEMRA cell (CD45RA$^+$CCR7$^-$) and CM T cell (CD45RA$^-$CCR7$^+$). Statistical differences were determined with one-way ANOVA for multiple variable comparisons.

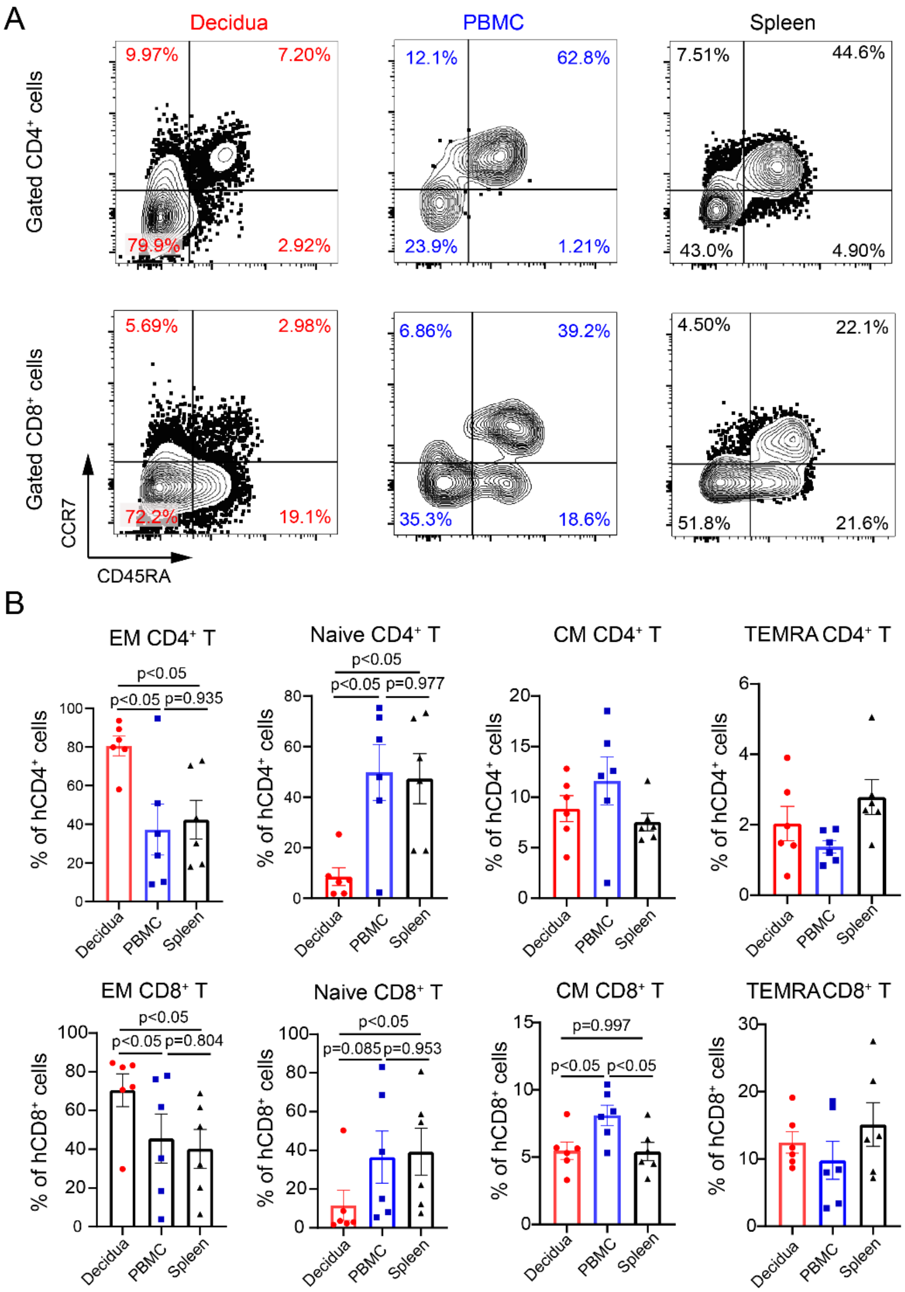

Figure S3. Determination of human immune subset composition in pregnant HIS mice made by mating with BALB/c male at E14.5. (**A**) Representative FCM profiles of CD45RA and CCR7 expression in human CD4$^+$ and CD8$^+$ T cells. (**B**) Summarized results (mean ± SEM; n = 6) of the percentages of EM T cell (CD45RA$^-$CCR7$^-$), Naive T cell (CD45RA$^+$CCR7$^+$), TEMRA cell (CD45RA$^+$CCR7$^-$) and CM T cell (CD45RA$^-$CCR7$^+$). Statistical differences were determined with one-way ANOVA for multiple variable comparisons.

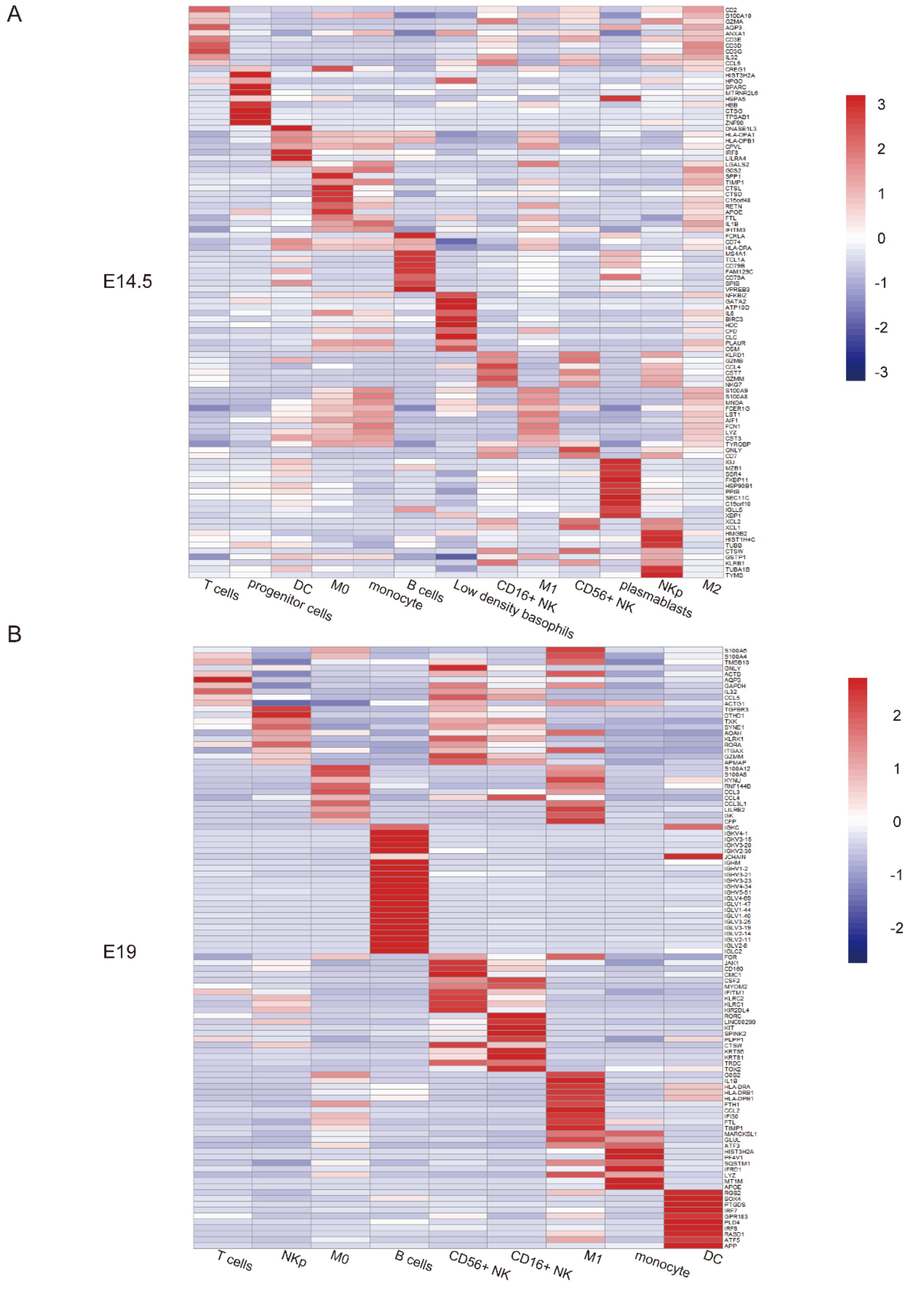

Figure S4. Heatmap showing expression levels of TOP 10 specific markers in each cell cluster at E14.5 (A) and E19 (B).

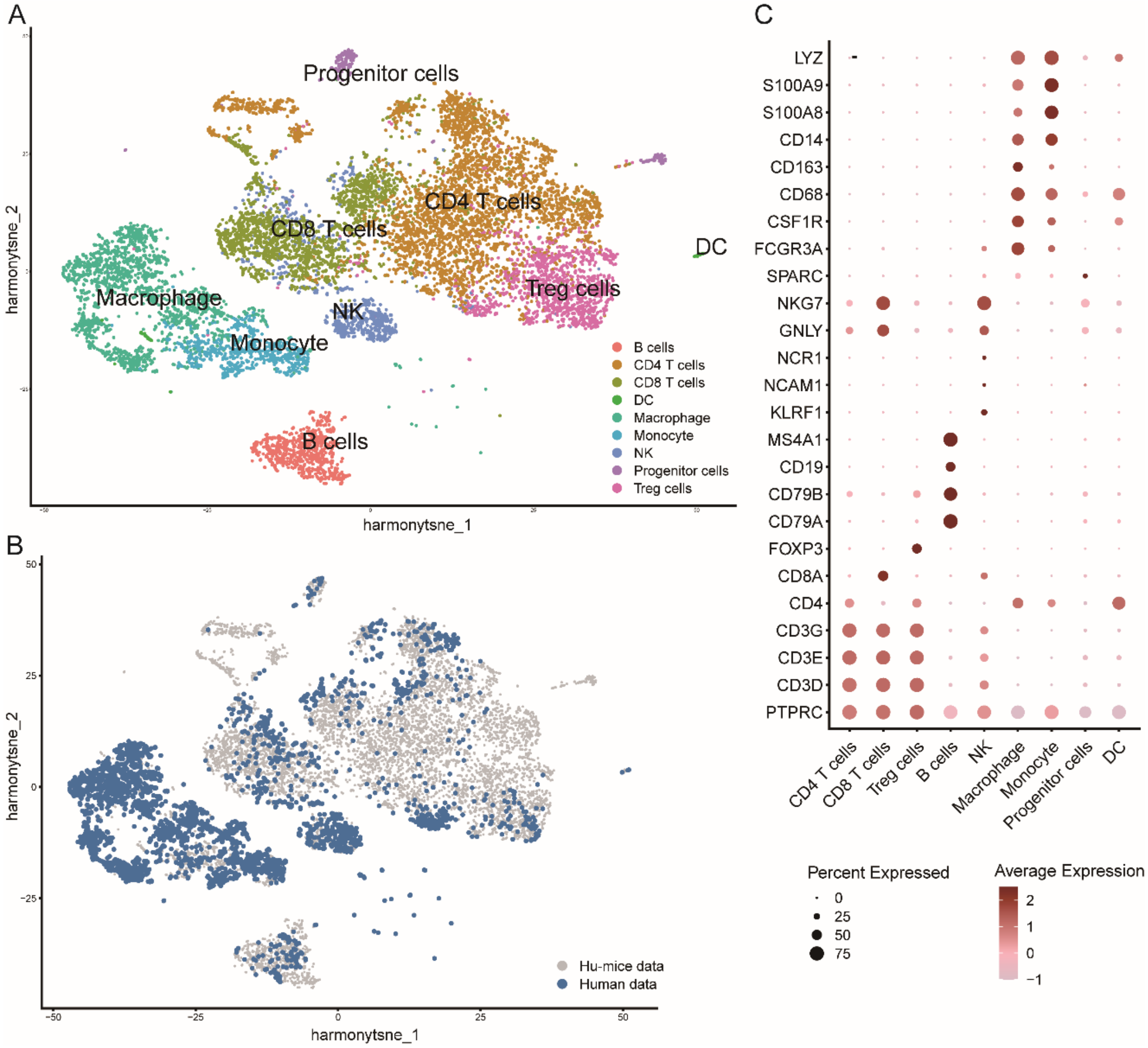

Figure S5. Integration analysis of human immune cell composition in HIS mouse decidua and the human counterpart. (A) Main immune cell types at the maternal-fetal interface of E14.5 HIS mice and human being (20-34 week). **(B)** t-SNE plots about the cell origin. **(C)** Dot plot of main immune cell types.

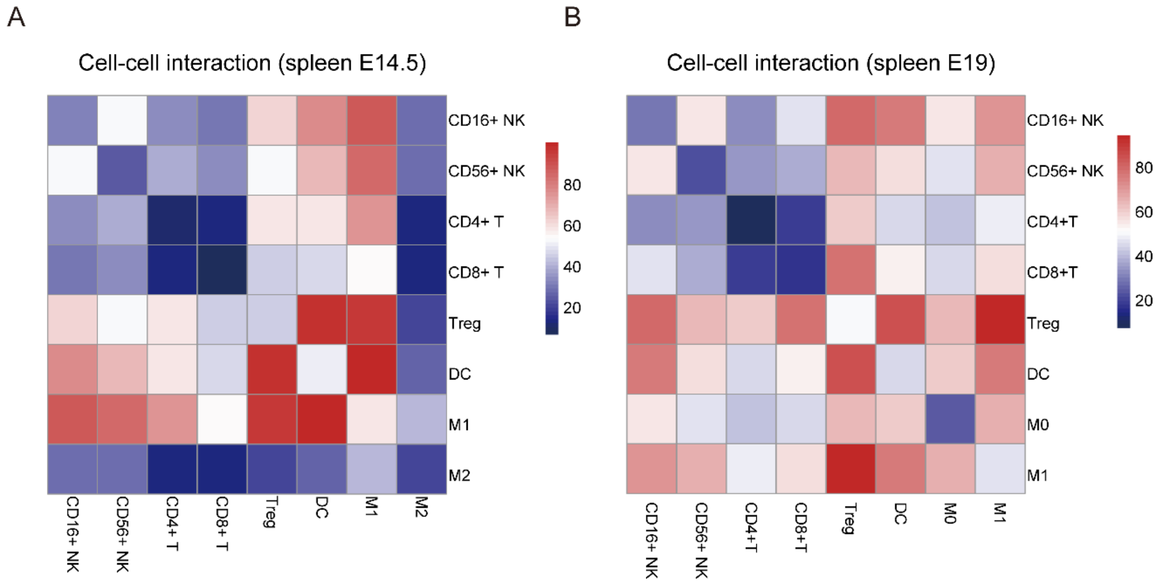

Figure S6. Cell-cell interactions examination for human immune subsets in spleen at E14.5 (A) and E19 (B).

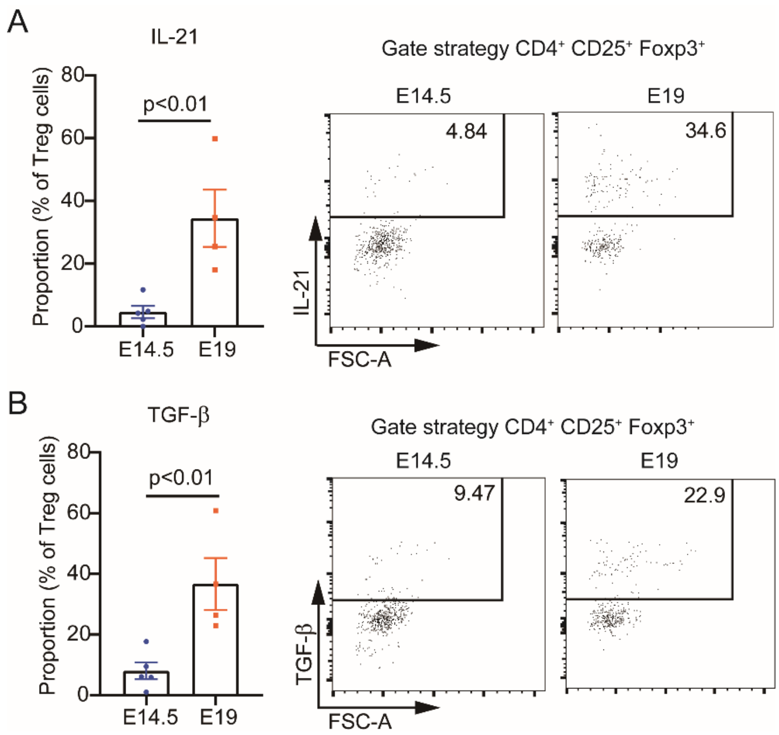

Figure S7. The protein expression of IL-21 and TGF-β in CD4$^+$ CD25$^+$ Foxp3$^+$ Treg cells. (A) Ratios (mean ± SEM) of IL-21$^+$ Treg cells in decidua at E14.5 (n=5) and E19 (n=4). Right side is representative flow cytometric profiles. (B) Ratios (mean ± SEM) of TGF-β$^+$ Treg cells in decidua at E14.5 (n=5) and E19 (n=4). Right side is representative flow cytometric profiles. Significance was determined by unpaired t-test.

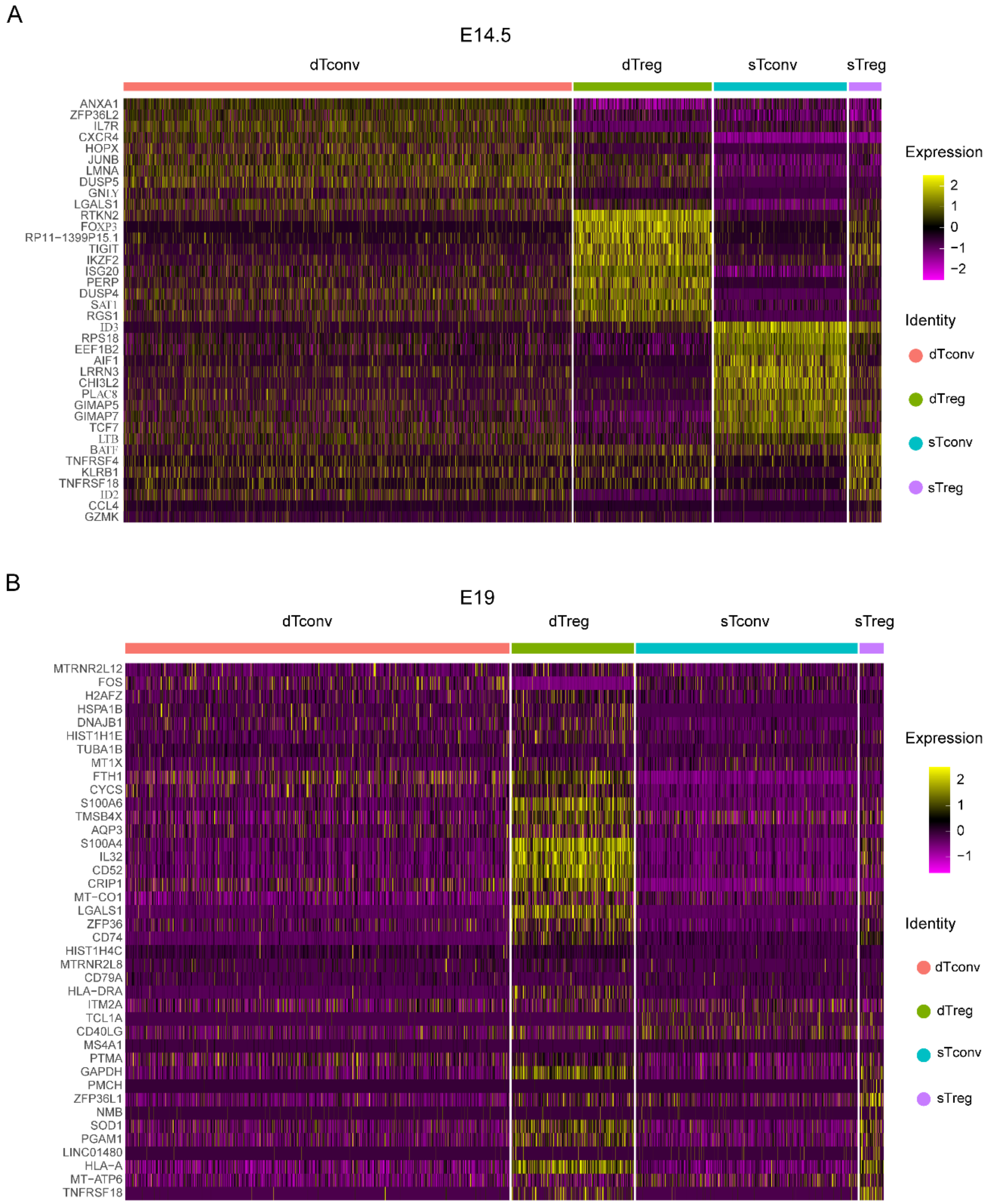

Figure S8. Heatmaps show the expression levels of top10 markers in Treg cells and Tconv cells of decidua and spleen at E14.5 (A) and E19 (B).

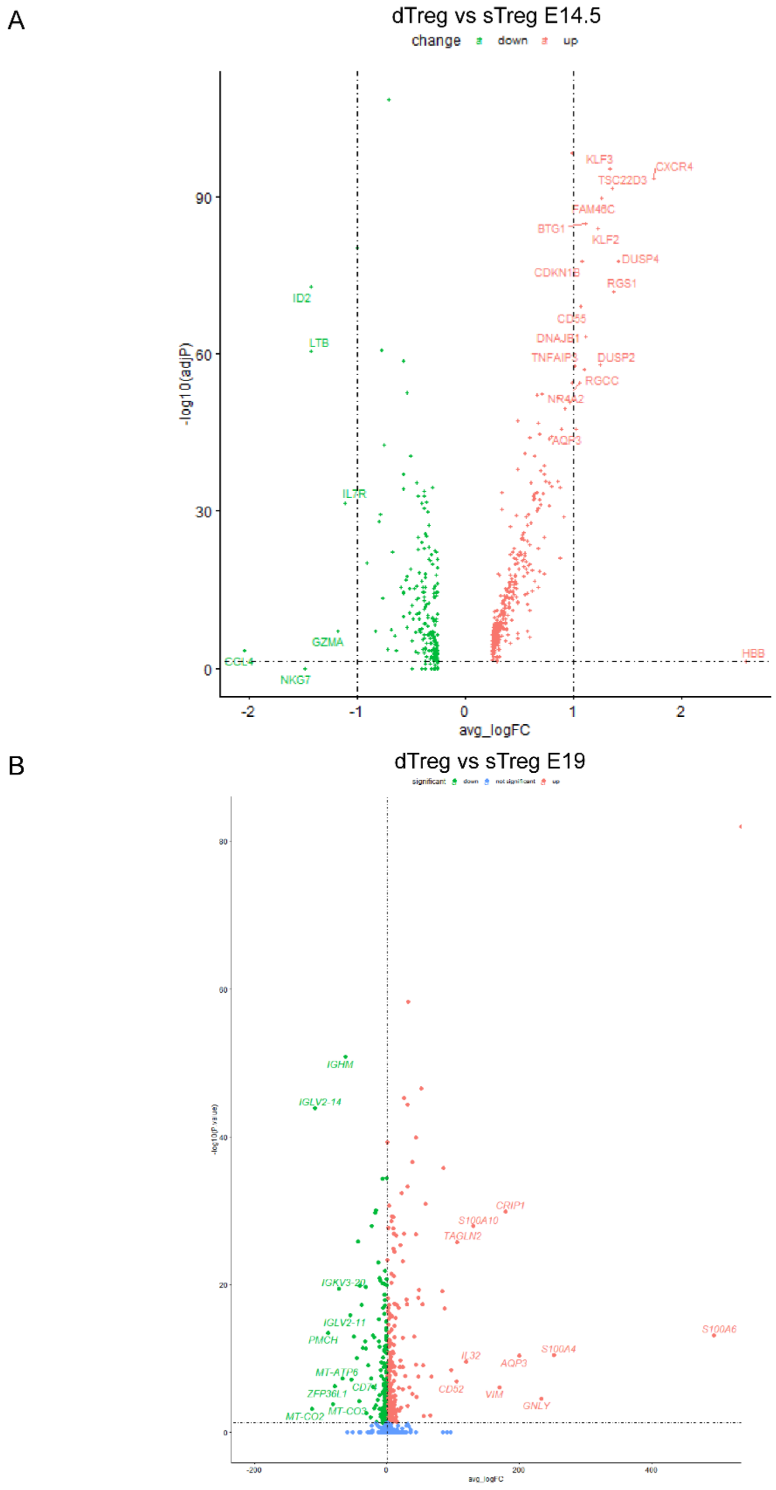

Figure S9. Volcano plots of differential gene expression between dTreg cells and sTreg cells at E14.5 (A) and E19 (B).

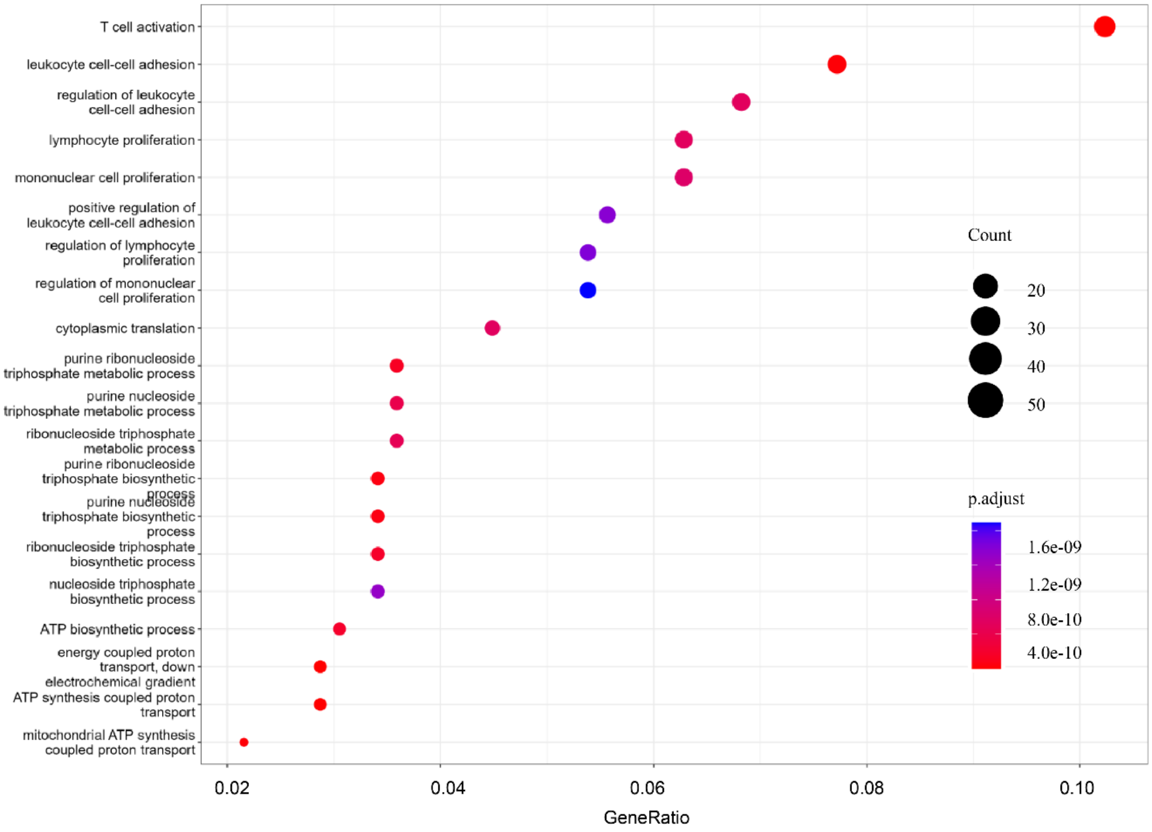

Figure S10. Gene set enrichment analysis was performed using the GO tool for significantly upregulated genes in dTreg cells at E19 relative to E14.5.

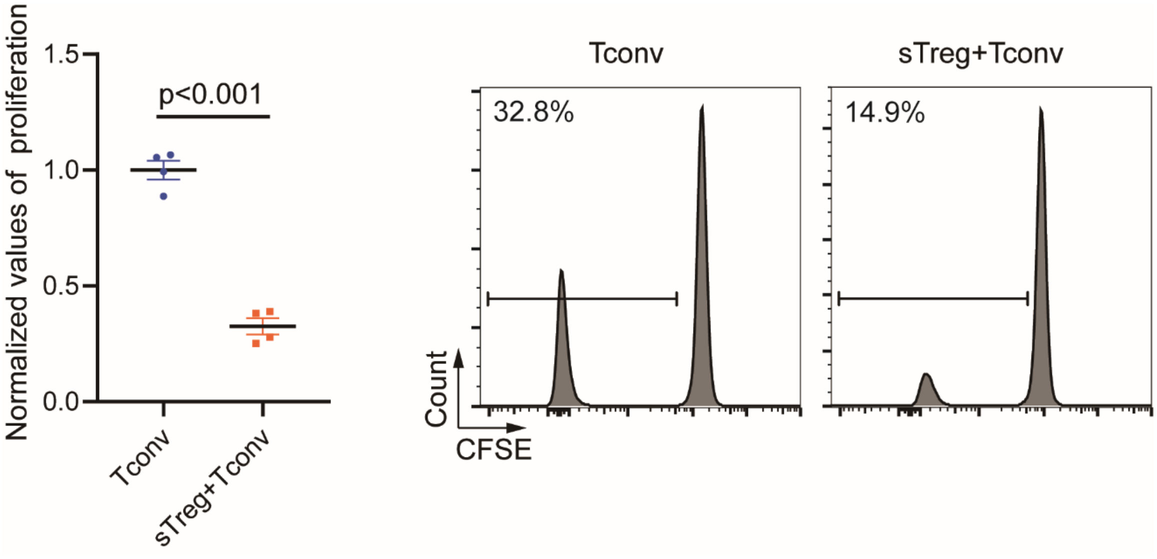

Figure S11. Summarized data (left; mean ± SEM) of the immune inhibitory effect of human sTreg cells to human sTconv cell proliferation in the presence of 30 Gy irradiated BALB/c splenic cells. Representative FCM profiles (right) of Tconv cell proliferation. Treg : Tconv = 1 : 2, BALB/c stimulator cells : Tconv = 1 : 2. Significance was determined by unpaired t-test.

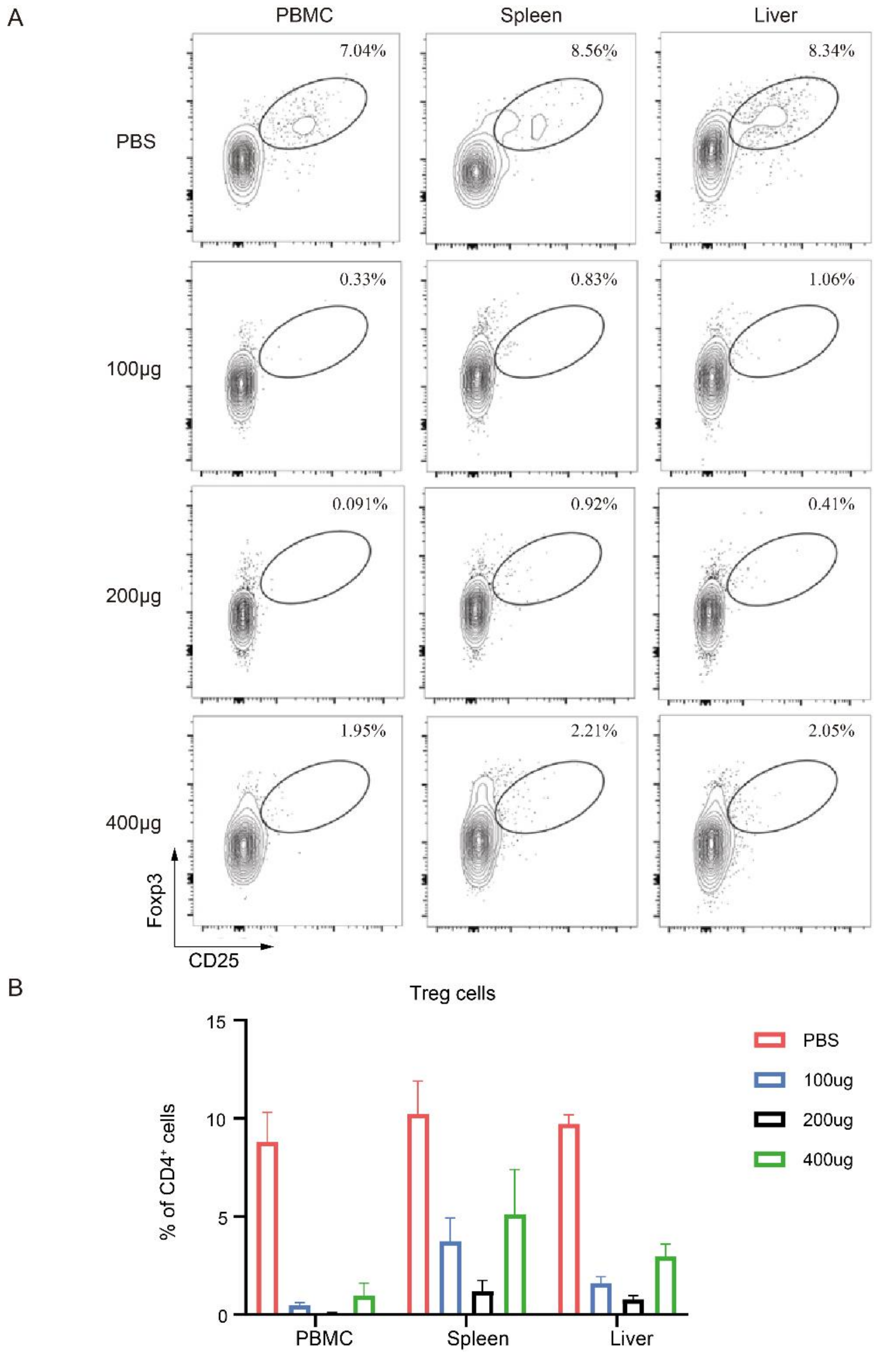

Figure S12. **Dosage tests of anti-CD25 mAb for Treg cell reduction in humanized mice.** Different dosages of anti-CD25 mAb (100 μg or 200 μg or 400 μg) or same volume of PBS was i.p. injected into non-pregnant HIS mice at week 10 after humanization. The mice were euthanized 6 days later for examination. (**A**) Representative FCM profiles and (**B**) percentages (n =3; mean ± SEM) of Treg cells in PBMC, spleen and liver were shown.

Table S1. (Antibody information for FCM)

**Antibodies**	**Fluorophore**	**Company**	**Catalog number**
Anti-human CD45	APC/Cy7	Biolegend	304014
Anti-human CD3	PE/Cy7	BD	557851
Anti-human CD4	PE	Biolegend	300508
Anti-human CD4	FITC	eBiosciecne	11-0048-42
Anti-human CD8	BV605	BD	564116
Anti-human CD45RA	FITC	BD	555488
Anti-human CCR7	APC	Biolegend	353214
Anti-human CD25	PE	Biolegend	356134
Anti-human Foxp3	PE/Cy5	eBiosciecne	15-4776-42
Anti-human CD19	APC	Biolegend	302212
Anti-human CD20	PE/Cy7	Biolegend	302312
Anti-human CD33	BV421	Biolegend	303416
Anti-human CD14	FITC	Biolegend	301804
Anti-human HLA-DR	PE/Cy7	Biolegend	307616

Anti-human CD80	BV605	BD	563315
Anti-human CD206	AF700	Biolegend	321132
Anti-human CD56	FITC	Biolegend	318304
Anti-human CD16	Percp/Cy5.5	Biolegend	302028
Anti-human CD49a	AF647	Biolegend	328310
Anti-human CD103	PE	BD	550260
Anti-mouse CD45	Pacific Blue	Biolegend	103126
Anti-human Helios	PE/Cy7	Biolegend	137236
Anti-human IL-21	PE	BD	560463
Anti-human TGF-β1	FITC	Biolegend	349606
Anti-mouse Ter119	PE/Dazzle 594	Biolegend	116244

Table S2. (Antibody information for IHC).

**Antibody**	**Company**	**Catalog number**
Anti-human CD45	DAKO	M070101
Anti-human CD4	ABclonal	A10787

REFERENCES

1. Novershtern N, et al. Densely interconnected transcriptional circuits control cell states in human hematopoiesis. *Cell.* 2011;144(2):296-309.
2. Monaco G, et al. RNA-Seq Signatures Normalized by mRNA Abundance Allow Absolute Deconvolution of Human Immune Cell Types. *Cell Rep.* 2019;26(6): 1627-1640.
3. Schmiedel BJ, et al. Impact of Genetic Polymorphisms on Human Immune Cell Gene Expression. *Cell.* 2018;175(6): 1701-1705.
4. Yu G, et al. clusterProfiler: an R package for comparing biological themes among gene clusters. *OMICS.* 2012;16(5):284-287.
5. Efremova M, et al. CellPhoneDB: inferring cell-cell communication from combined expression of multi-subunit ligand-receptor complexes. *Nat Protoc.* 2020;15(4):1484-1506.
